# Assessing the Association Between T2DM, Rheumatoid Arthritis, and Dipeptidyl Peptidase‐4 Inhibitors: Insights From Epidemiology, Meta‐Analysis, and Mendelian Randomization Study

**DOI:** 10.1155/jdr/9011744

**Published:** 2026-03-11

**Authors:** Lirong Zhang, Lan Lin, Jingting Wang, Yixiao Zhu, Jiaqin Cai, Hong Sun, XiaoXia Wei

**Affiliations:** ^1^ Department of Pharmacy, Fuzhou University Affiliated Provincial Hospital, Fuzhou, China; ^2^ School of Pharmacy, Fujian Medical University, Fuzhou, China, fjmu.edu.cn; ^3^ Information Management Center, Fuzhou University Affiliated Provincial Hospital, Fuzhou, China; ^4^ Fujian Provincial Key Laboratory of Medical Big Data Engineering, Fuzhou, China

**Keywords:** dipeptidyl peptidase-4 inhibitor, drug-targeted and mediation mendelian randomization study, national health and nutrition examination survey, rheumatoid arthritis, Type 2 diabetes mellitus

## Abstract

**Objective:**

This study explores the association between Type 2 diabetes mellitus (T2DM), dipeptidyl peptidase‐4 inhibitors (DPP4i), and rheumatoid arthritis (RA).

**Methods:**

We conducted a comprehensive analysis using data from the National Health and Nutrition Examination Survey (NHANES), existing studies, and genome‐wide association studies (GWAS). Weighted logistic regression was employed to investigate the association between T2DM and RA. A meta‐analysis was performed to examine the relationship between DPP4i use and the risk of RA. Additionally, a drug target‐mediation mendelian randomization (MR) study was conducted to evaluate the causal relationship between DPP4i and RA, as well as potential pathway mechanisms.

**Results:**

The NHANES analysis revealed T2DM was associated with RA (OR = 1.31). The meta‐analysis, which included 12 studies, indicated a reduced risk of RA among DPP4i users (RR = 0.63). MR analysis demonstrated that DPP4i use was associated with a decreased risk of RA (OR = 0.84). Mediation MR analysis suggested that DPP4i might influence RA development through immune factors such as CD14 + CD16+ monocytes, CXCL11, and IL‐2 receptors.

**Conclusions:**

This study confirmed a significant association between T2DM and RA and further revealed that DPP4i might reduce RA risk through inflammation and immune modulation. Further studies are needed to confirm these findings.

## 1. Introduction

Rheumatoid arthritis (RA) is a chronic systemic autoimmune disease characterized by synovial inflammation, joint destruction, and dysregulated systemic immune responses. [1] The etiology of RA remains incompletely understood; however, genetic predisposition, environmental factors, and aberrant activation of the immune system are known to play critical roles in its pathogenesis. [2] In recent years, there has been an increasing emphasis on the role of metabolic disorders in the development of RA, specifically focusing on Type 2 diabetes mellitus (T2DM). [3] Several epidemiological studies have revealed the potential positive association between T2DM and RA. [4–6] This indicates the possibility that the two conditions may involve shared inflammatory mechanisms, including insulin resistance, chronic low‐grade inflammation, and immune cell dysfunction. [7] However, a cohort study conducted by Jin et al. reported a negative association between T2DM and RA. [8]

Currently, a wide range of therapeutic options are available for the treatment of T2DM; dipeptidyl peptidase‐4 inhibitors (DPP4i) have garnered particular attention due to their potential anti‐inflammatory and immunomodulatory effects. [9] DPP4, also known as CD26, is a serine protease that is widely expressed in various tissues and plays a crucial role in the immune system. It regulates T cell activation, cytokine secretion, and immune cell infiltration. [10] Previous review articles have summarized evidence suggesting that DPP4i not only regulate blood glucose by inhibiting DPP4 activity but might also influence the development and progression of inflammatory diseases through their immunomodulatory effects. [11] On this basis, preliminary studies have explored the potential association between DPP4i and RA, yielding heterogeneous findings. [12–16] Several observational studies indicated that the use of DPP4i may be associated with a reduced risk of RA. [12, 15, 16] For example, cohort studies reported that T2DM patients treated with DPP4i had a lower incidence of RA compared with those treated with other antidiabetic medications, indicating a possible immunomodulatory role of DPP4i. [12] In contrast, other observational studies have failed to identify a significant association between DPP4i use and RA risk, [13] and a limited number of case reports have described the development of RA following DPP4i exposure. [14] The results of the meta‐analyses also showed significant differences. A meta‐analysis including four cohort studies reported that the use of DPP4i was associated with a reduced risk of RA (RR = 0.72, 95% CI: 0.54–0.96), though heterogeneity was high in this study (*I* ^2^ = 75*%*). [17] A more recent meta‐analysis incorporating both cohort studies and randomized controlled trials (RCTs) reported contradictory findings, indicating that DPP4i do not significantly affect the risk of RA (RR = 0.96, 95% CI: 0.69–1.32). [18] Notably, this study did not strictly distinguish between different types of arthritis, osteoarthritis, and RA. An overview of representative epidemiological studies exploring the associations between T2DM or DPP4i and RA is presented in Table S1.

Furthermore, the mechanism by which DPP4i influence RA remains unclear. The development of RA involves complex immune regulatory processes, including interactions among various immune cells, such as T cells, B cells, and monocyte–macrophages, as well as inflammatory cytokines, including tumor necrosis factor alpha (TNF‐*α*), interleukin‐6 (IL‐6), and interleukin‐17 (IL‐17). [19–22] Among these pathways, T cell–mediated immune responses play a central role in RA pathogenesis, and interleukin‐2 (IL‐2) signaling through IL receptor (IL‐2R) is critical for regulating T cell activation, proliferation, and immune tolerance. [23, 24] DPP4i may influence the development and progression of RA by modulating the levels of immune cells and inflammatory cytokines. [25–29] However, current research on whether DPP4i can reduce RA risk through immune‐mediated mechanisms remains limited, and systematic studies focusing on specific immune pathways and molecular mechanisms are still lacking.

Current research on the association between T2DM, DPP4i, and RA remains conflicting, with most studies relying on retrospective data, which are limited by confounding factors, making it difficult to establish a causal association between DPP4i and RA. [12–17] Therefore, larger‐scale studies and more rigorous methodologies are needed to further investigate the association and underlying mechanisms among T2DM, DPP4i, and RA. The National Health and Nutrition Examination Survey (NHANES), conducted by the National Center for Health Statistics, is a nationally representative, ongoing survey that collects extensive health and nutrition data from thousands of participants annually, providing essential support for comprehensive epidemiological research and generalizable findings. [30] Meta‐analysis is a method of quantitatively synthesizing existing studies to derive conclusions that are both comprehensive and reliable. [31, 32] The timely updating of results enhances their reliability and more effectively addresses clinical controversies. Mendelian randomization (MR) studies utilize genetic variants as instrumental variables (IVs), thereby effectively addressing confounding factors and the limitations of causal inference in observational studies. To some extent, MR can approximate the causal inference strength of RCTs. [33] Drug‐target MR can be used to investigate the causal association between a specific drug and a disease, providing valuable insights for drug development and personalized treatment. [34] Mediation MR is used to assess potential mediators in a causal pathway, exploring how genetic variants influence outcomes through intermediary variables. This approach helps to identify the specific mechanisms underlying drug effects. [35]

Therefore, we integrated NHANES database analysis, meta‐analysis, and drug target‐mediated MR studies to provide more reliable evidence on the association between T2DM, DPP4i, and RA from multiple perspectives, with a particular focus on the role of DPP4i in the progression of RA.

## 2. Methods

In this study, we utilized publicly available data that had received appropriate ethical approvals. As the study was based on aggregated, deidentified genetic data, institutional review board approval and informed consent were not required. This study adhered to the Strengthening the Reporting of Observational Studies in Epidemiology using Mendelian Randomization (STROBE‐MR) guidelines for transparent and standardized reporting [36]

## 3. NHANES Analysis

### 3.1. Study Population

In this study, we collected data from individuals who participated in the NHANES survey between 1999 and 2023. To ensure the accuracy of the analysis, we excluded individuals with unknown T2DM diagnosis and those diagnosed with other types of arthritis. Participants younger than 20 years old and pregnant women were excluded. We also removed individuals with missing or incomplete data on key covariates, including education level, health insurance status, body mass index (BMI), smoking history, alcohol consumption, and hypertension history. T2DM was defined as a physician‐diagnosed diabetes condition managed with antidiabetic medications, with Type 1 diabetes (T1DM) patients excluded. T1DM was defined as diabetes diagnosed before the age of 20 and requiring insulin therapy within 1 year of diagnosis. RA diagnosis was based on self‐reported data from the questionnaire. Participants were first asked, “Has a doctor ever told you that you have arthritis?” If the response was “yes”, they were further asked, “What type of arthritis do you have?” Those who reported RA were classified as having RA. Definitions and grading criteria for other relevant variables are attached in Table S2. Weighted *t*‐tests and chi‐square tests were used to compare group differences, whereas weighted logistic regression models were employed to assess the association between T2DM and RA, adjusting for demographic, socioeconomic, and clinical variables. Subgroup and interaction analyses were conducted to explore potential influencing factors.

## 4. Meta‐Analysis

We systematically searched six major databases, including PubMed, Embase, Cochrane Library, Web of Science, Cochrane Central Register of Controlled Trials, and Scopus. The search covered the period from database inception to December 31, 2024. The search terms included “Dipeptidyl Peptidase‐4 Inhibitors” and “rheumatoid arthritis”. The detailed search strategy was provided in Table S3. We included only RCTs and cohort studies investigating the association between DPP4i and RA. The specific inclusion and exclusion criteria are detailed in Table S4. Two researchers independently screened studies meeting the inclusion criteria and extracted the relevant data. Extracted information included basic study details (first author, publication year, country, and study title), study design and key quality assessment indicators, baseline characteristics of the intervention and control groups (including gender, age, and ethnicity), data sources, and total sample size and event counts in both the intervention and control groups. Any discrepancies were resolved through discussion with a third reviewer. The Cochrane Risk of Bias tool and the Newcastle‐Ottawa Scale (NOS) were used to assess the quality of RCTs and cohort studies, respectively. Fixed‐effects or random‐effects models were applied to calculate relative risk (RR), with model selection based on heterogeneity (*I* ^2^ statistic). Subgroup and sensitivity analyses were conducted to identify potential sources of heterogeneity, whereas Egger′s test, Begg′s test, and funnel plots were used to assess publication bias.

## 5. MR Study

The target of DPP4i is DPP4. [37] To minimize population stratification bias, this study was conducted in a European‐ancestry population. The data used for the MR analysis are summarized in Table S5.

The selection of IVs followed three fundamental principles: The selected single nucleotide polymorphisms (SNPs) should be strongly associated with the exposure, influence the outcome only through the exposure, and remain independent of potential confounders [38] To ensure compliance with these principles, we extracted cis‐acting SNPs within a 500 kb region surrounding the DPP4 gene from the eQTL database, applying thresholds of *p* < 5 × 10^−8^, minor allele frequency (MAF) > 1*%*, and false discovery rate (FDR) < 0.05. To minimize linkage disequilibrium (LD) bias, independent SNPs were selected using LD clumping (*r* ^2^ < 0.3, 500 kb). Additionally, the association between each DPP4 variant and glycated hemoglobin (HbA1c) levels was assessed, and SNPs with an F − statistic < 10 were excluded to remove weak instrument bias. We also conducted a colocalization analysis between DPP4 and HbA1c, using a posterior probability threshold of > 80% as evidence of colocalization. The final IVs obtained through this process were used as proxies for DPP4i.

This MR study consisted of two main components. In the first step, a two‐sample MR analysis was conducted to evaluate the causal association of DPP4i on RA. In the second step, mediation MR was used to evaluate the mediating role of 91 inflammatory factors and 731 immune cells in the DPP4 inhibitor and RA pathways. In the mediation analysis, we first identified immune cells and inflammatory cytokines that had a causal association on RA, then further evaluated those that were also causally associated with DPP4i. The product of coefficients method was used to estimate the mediation effect. In the MR analysis, the inverse‐variance weighted (IVW) method was the primary analysis method, as it assumes that more than 50% of the IVs are valid. The MR‐Egger regression and weighted median were used as supporting methods. MR results were reported as odds ratio (OR) with corresponding 95% CI. Sensitivity analyses included Cochran′s Q test, MR‐Egger‐intercept test, MR‐PRESSO, and leave‐one‐out analysis to assess heterogeneity, horizontal pleiotropy, and robustness. To visualize the results, scatter plots, forest plots, leave‐one‐out plots, and funnel plots were generated. All statistical analyses were conducted using R software (Version 4.3.1).

## 6. Result

### 6.1. NHANES Analysis Results

#### 6.1.1. Characteristics of Study Participants

The final study sample comprised 119,555 eligible participants (Figure S1). Patients with RA showed significantly different characteristics compared with non‐RA participants. RA participants were older (60.21 ± 0.28 years vs. 43.58 ± 0.22 years) and had a higher proportion of females (64% vs. 36%). Significant differences were also observed between the two groups in terms of race, education level, health insurance status, smoking, alcohol consumption, hypertension, and BMI (*p* < 0.05). Notably, the prevalence of T2DM among RA patients was 14%, more than twice that of non‐RA participants (5%) (Tables 1 and S6).

**Table 1:** Baseline characteristics of RA group versus the non‐RA group.

Characteristics	Overall	RA	Non‐RA	*p* value
Age (years)	47.64 ± 0.2292	60.21 ± 0.28	43.58 ± 0.22	< 0.0001
Gender (%)				< 0.0001
Male	6572 (0.42)	1517 (0.36)	5055 (0.44)	
Female	8490 (0.58)	2634 (0.64)	5856 (0.56)	
Race (%)				< 0.0001
Hispanic	336 (0.12)	700 (0.07)	2636 (0.13)	
Non‐Hispanic	11726 (0.88)	3451 (0.93)	8275 (0.87)	
Education level (%)				< 0.0001
Less than high school	3278 (0.13)	1016 (0.15)	2262 (0.12)	
High school or GED	3108 (0.21)	933 (0.24)	2175 (0.20)	
Above high school	8676 (0.66)	2202 (0.61)	6474 (0.68)	
Health insurance				< 0.0001
Yes	12346 (0.85)	3787 (0.92)	8559 (0.83)	
No	2716 (0.15)	364 (0.08)	2352 (0.17)	
Smoking				< 0.0001
Yes	6772 (0.46)	2183 (0.54)	4589 (0.43)	
No	8290 (0.54)	1968 (0.46)	6322 (0.57)	
Alcohol (%)				0.003
Yes	2432 (0.15)	773 (0.17)	1659 (0.15)	
No	12630 (0.85)	3378 (0.83)	9252 (0.85)	
BMI (kg/m^2^)	28.76 ± 0.09	30.71 ± 0.17	28.12 ± 0.09	< 0.0001
T2DM (%)				< 0.0001
Yes	1456 (0.07)	728 (0.14)	728 (0.05)	
No	13606 (0.93)	3423 (0.86)	10183 (0.95)	
Hypertension (%)				< 0.0001
Yes	5161 (0.30)	2344 (0.52)	2817 (0.23)	
No	9901 (0.70)	1807 (0.48)	8094 (0.77)	

Abbreviations: BMI, body mass index; GED, general educational development; RA, rheumatoid arthritis; T2DM, Type 2 diabetes mellitus.

#### 6.1.2. Weighted Logistic Regression Analysis Revealed the Association Between T2DM and RA

**Figure 1:**
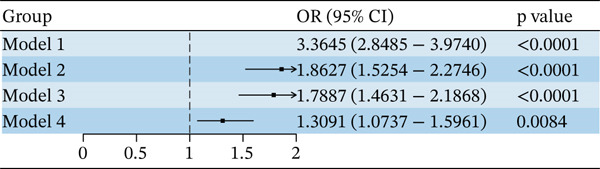
Association between T2DM and RA. Abbreviations: Model 1: No covariates were adjusted; Model 2: adjusted for age, gender, and race; Model 3: further adjusted for health insurance and education level; and Model 4: further adjusted for BMI, smoking, alcohol, and hypertension.

**Figure 2:**
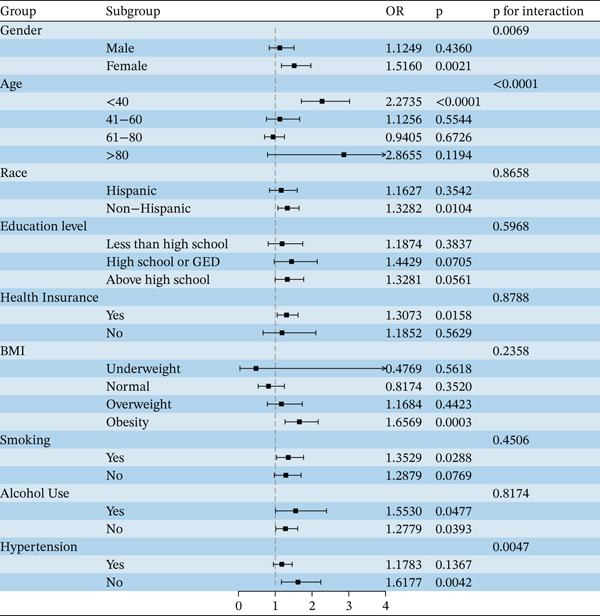
Subgroup analysis of the association between T2DM and RA.

Figure 1 presented the results of the weighted logistic regression analysis assessing the association between T2DM and RA before and after adjustment for covariates. Overall, T2DM was significantly associated with RA (OR = 3.36, 95% CI: 2.85–3.97). After adjusting for demographic and socioeconomic factors, smoking, alcohol consumption, hypertension status, and BMI, this association remained statistically significant (OR = 1.31, 95% CI: 1.07–1.60). Subgroup analyses further revealed that the association between T2DM and RA remained significant among women, individuals younger than 40 years old, non‐Hispanic participants, those with health insurance, obese individuals, smokers, and drinkers. Age and gender affected the association between DPP4i and RA (*p* for interaction < 0.05). Notably, the presence of hypertension did not change the association between DPP4i and RA (Figure 2).

### 6.2. Meta‐Analysis Results

The search strategy initially identified 974 potentially relevant studies. After screening, we ultimately included 12 studies, comprising eight RCTs and four cohort studies. (Figure S2) Most of the studies were conducted in multiple regions, whereas others were conducted in the United States, China, South Korea, and Turkey. The average age of most participants was between 50 and 60 years, the sample size ranged from 245 to 1,147,784, and the follow‐up time ranged from 7 months to 8.3 years. (Table S7) In terms of quality assessment, RCTs were generally low risk. All included cohort studies had a NOS score of ≥ 7, indicating that all included studies were of moderate to high quality. (Table S8 and Figures S3 and S4).

**Figure 3:**
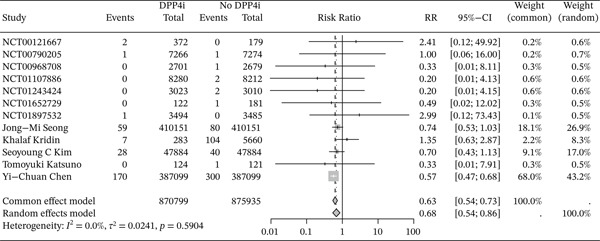
Forest plots were used to assess the risk of RA in those DPP4i users versus those not using DPP4i.

**Figure 4:**
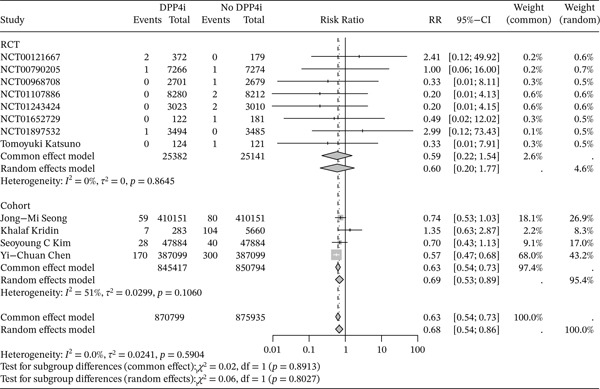
Forest plots were used to assess the risk of RA in those DPP4i users versus nonusers in different study designs.

**Figure 5:**
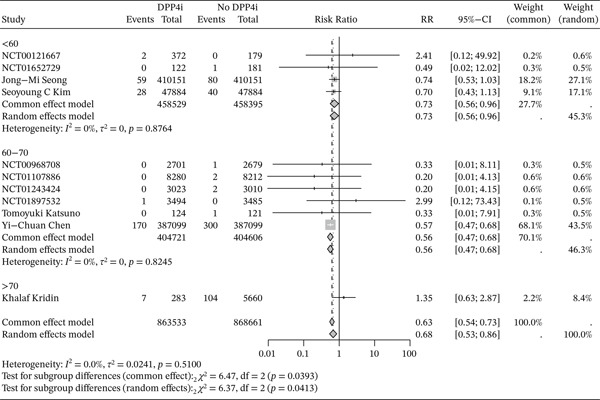
Forest plots were used to assess the risk of RA in those DPP4i users versus nonusers in different ages.

This study had low heterogeneity (*I* ^2^ = 0*%*, *p* = 0.59), thus we used a fixed‐effects model to analyze. The results demonstrated a negative association between DPP4i use and RA risk (RR = 0.63, 95% CI: 0.54–0.73). (Figure 3) Further subgroup analyses were conducted based on study design and age. In cohort studies, individuals under the age of 70 who were treated with DPP4i, the risk of RA was significantly lower than in those who did not take DPP4i. DPP4i also showed a similar protective effect against RA in RCTs. (Figures 4 and 5).) Sensitivity analyses further confirmed the robustness of the findings. No evidence of publication bias was detected using Egger′s test (*p* = 0.49) and Begg′s test (*p* = 0.84), and the funnel plots appeared largely symmetrical. (Figures S5 and S6)

### 6.3. MR Study Results

#### 6.3.1. Drug‐Target MR Analysis Results

**Figure 6:**
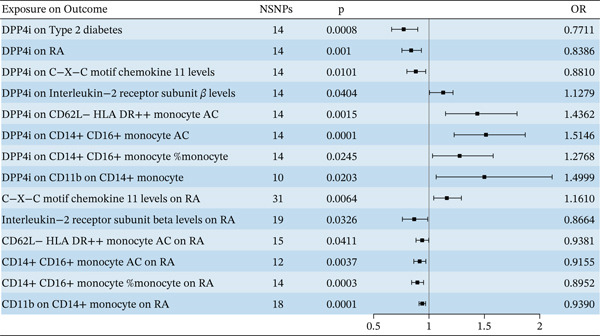
MR estimates derived from the IVW method to assess the causal effect between DPP4i, inflammatory factors, immune cells, and RA.

Based on the IVs selection criteria, 14 SNPs were identified as genetic proxies for DPP4i effects. All SNPs had F‐statistics greater than 10, eliminating the influence of weak instruments (Table S9). The IVW model analysis demonstrated a causal association between DPP4i and RA, indicating that for each SD increase in DPP4i, the risk of RA decreased by 16% (OR = 0.84, 95% CI: 0.76–0.93) (Figure 6). The MR‐Egger and weighted median yielded consistent results (Table S10). Sensitivity analyses detected no evidence of heterogeneity or horizontal pleiotropy (*p* values of Cochran Q test, MR‐PRESSO, and MR‐Egger intercept test were all > 0.05) (Table S11). Leave‐one‐out analysis revealed that no single SNP had an obvious influence on the causal inference (Figure S7).

#### 6.3.2. Mediation MR Analysis Results

We estimated the effects of 91 inflammatory cytokines and 731 immune cell types on RA and then identified 15 inflammatory cytokines and 11 immune cell types that exhibited a causal association with RA. Further analysis of the association between DPP4i and these inflammatory cytokines and immune cells revealed that DPP4i was causally associated with eight inflammatory cytokines and 11 immune cell types (Table S12). Mediation effect analysis and mediation proportion calculations were conducted for the identified inflammatory cytokines and immune cells. The results indicated that two inflammatory cytokines and four immune cell types acted as mediators in the association between DPP4i and RA (Figure 5). As shown in Table 2, DPP4i exerted an indirect effect on RA risk through CD14 + CD16+ monocyte AC, CD14 + CD16+ monocyte % monocyte, CD11b on CD14+ monocyte, CD62L − HLA − DR + + monocyte AC, C‐X‐C motif chemokine 11 (CXCL11) levels, and interleukin‐2 receptor subunit beta (IL‐2R*β*) levels. The proportions of the total effect mediated by these factors were 2%, 15%, 14%, 13%, 11%, and 10%, respectively (Table 2).

**Table 2:** The mediation proportion of mediators in the causal relationship between DPP4i and RA.

Trait	Beta1	Beta2	Beta	Intermediate ratio	95% CI
CD14 + CD16+ monocyte AC	0.42	−0.09	−0.18	2%	[−0.07, −0.01]
CD14 + CD16+ monocyte % monocyte	0.24	−0.11	−0.18	15%	[−0.06, −0.002]
CD11b on CD14+ monocyte	0.41	−0.06	−0.18	14%	[−0.05, −0.003]
CD62L − HLA DR + + monocyte AC	0.36	−0.06	−0.18	13%	[0.0006, 0.05]
C‐X‐C motif chemokine 11 levels	−0.13	0.15	−0.18	11%	[−0.04, −0.002]
Interleukin‐2 receptor subunit beta levels	0.12	−0.14	−0.18	10%	[‐0.04, −0.0001]

## 7. Discussion

In this study, we are aimed at elucidating the complex association between T2DM, DPP4i, and RA, with a particular focus on the potential causal association and underlying mechanisms between DPP4i and RA. Our findings provided compelling evidence demonstrating that T2DM may be a risk factor for RA, and the protective effect of DPP4i on RA may be mediated through immune pathways.

The results of this study showed that the prevalence of T2DM in patients with RA was almost three times that of patients without RA. Even after adjusting for multiple confounding factors, the association between RA and T2DM remained robust. T2DM is characterized by a chronic inflammatory state and can lead to immune system dysregulation, including elevated levels of pro‐inflammatory cytokines such as TNF‐*α* and IL‐6, as well as imbalanced T cell regulation. [39, 40] These inflammatory and immune alterations may create a favorable environment for the development of RA. Notably, this study also found a strong association between T2DM and RA in individuals younger than 40 years old (OR = 2.27). Early‐onset T2DM is often accompanied by more severe metabolic abnormalities, such as insulin resistance and hyperinsulinemia, and is associated with an earlier and higher incidence of various complications. [41] Additionally, lifestyle factors such as obesity, unhealthy diet, and smoking may have a greater impact on younger individuals, further contributing to the increased risk of RA in early‐onset T2DM patients. [42] Previous studies on the association between diabetes and arthritis have shown conflicting results. An analysis of U.S. populations from 2009 to 2016 suggested that the burden of comorbidity between prediabetes and arthritis was higher in individuals aged 65 years and older. [43] However, a more recent study indicated that the association between diabetes and arthritis was negatively correlated with age. [6] These discrepancies further underscore the importance of developing personalized healthcare strategies to address the varying impacts of T2DM on RA. Hypertension is a common risk factor for both diabetes and RA. [44] Initially, we hypothesized that T2DM and hypertension would have a synergistic effect on RA risk. However, our findings revealed that hypertension did not affect the association between T2DM and RA. Due to the relatively small sample size of participants with hypertension in this study, its specific role in the impact of T2DM on RA should be interpreted with caution.

Our study also demonstrated that DPP4i are associated with a reduced risk of RA. DPP4 possesses three key functions related to inflammation regulation, including binding to adenosine deaminase, interacting with the extracellular matrix, and exerting exopeptidase activity. [45] Previous studies have demonstrated that modulating DPP4 activity can influence multiple immune pathways involved in RA pathogenesis. [46–52] For instance, sitagliptin (a type of DPP4i) has been shown to inhibit T helper cell 1 (Th1) and Th17 differentiation in a dose‐dependent manner in vitro, both of which are key immune components involved in RA pathogenesis. [48–50] Studies in DPP4‐knockout mice have demonstrated reduced levels of transforming growth factor‐*β*1 (TGF‐*β*1), a cytokine known to mediate systemic inflammation, induce fibroblast proliferation, and promote synovial hypertrophy in RA. [51, 52] Therefore, it is reasonable to speculate that DPP4i‐mediated inhibition of DPP4 may lead to similar immunomodulatory effects, ultimately reducing the inflammatory burden associated with RA. [46, 48, 51] These findings are consistent with the results of some previous observational studies. [12, 15] The effects of different DPP4i on RA may vary. Chen et al. reported that the risk of RA was significantly lower in linagliptin (a type of DPP4i) users compared with sitagliptin users (HR: 0.25, 95% CI: 0.11–0.59) [12] Conversely, Douros et al. conducted a large‐scale study based on the UK population, specifically investigating the association between DPP4i use and incident RA. Their findings indicated that, compared with other antidiabetic drugs, DPP4i were not associated with RA risk (HR: 1.0, 95% CI: 0.8–1.3), regardless of treatment duration or the specific type of DPP4i used. [13] Therefore, the protective effect of DPP4i on RA may be influenced by factors such as population characteristics, drug type, and treatment duration, necessitating further investigation to validate these findings.

Although multiple studies have investigated the anti‐inflammatory and immunomodulatory effects of DPP4i, there have been no experimental studies that have specifically examined the mechanistic pathways linking DPP4i to RA. Therefore, we conducted a mediation MR analysis, which revealed that DPP4i might reduce RA risk by modulating specific immune cells and inflammatory cytokines. Studies have shown that CD14 + CD16+ monocytes are highly enriched in RA synovial tissue and can promote the release of pro‐inflammatory cytokines, such as TNF‐*α* and IL‐1*β*, thereby exacerbating RA‐associated inflammatory responses. [53, 54] This study found that DPP4i can modulate the expression of CD14 + CD16+ monocytes, suggesting that it may influence RA progression by regulating the infiltration of pro‐inflammatory monocytes. Meanwhile, CXCL11, a chemokine significantly elevated in the serum of RA patients, plays a key role in promoting T cell and monocyte recruitment, thereby exacerbating synovial inflammation. [55–57] This study suggested that DPP4i might reduce RA risk by inhibiting CXCL11 expression, thereby limiting the infiltration of inflammatory cells and mitigating synovial inflammation. The IL‐2/IL‐2R plays a crucial role in immune regulation in RA; abnormal IL‐2R expression may lead to excessive T cell activation, thereby exacerbating RA‐associated immunopathology. [58] This study further demonstrated that DPP4i might modulate IL‐2R signaling, potentially contributing to the restoration of immune balance in RA. These research results indicated that DPP4i might play a role in RA immune regulation, providing a new research direction for the potential therapeutic applications of DPP4i in autoimmune diseases.

This study has several strengths. Firstly, this study integrated NHANES data, meta‐analysis, and MR analysis to address the limitations inherent in any single research method, thereby enhancing the reliability of the research conclusions. Secondly, it provides strong causal inference. MR analysis, which uses genetic variants as IVs, effectively reduces confounding and reverse causation, providing robust evidence for the potential impact of DPP4i on RA risk. Finally, this study not only assessed the relationship between DPP4i and RA but also analyzed the role of immune cells and inflammatory factors between DPP4i and RA. This analysis elucidated the potential biological mechanisms underlying DPP4i, thereby offering new insights for the prevention and treatment of RA in the future. Despite these strengths, certain limitations must be acknowledged. Firstly, NHANES data do not include detailed information on diabetes medication use. Although we identified a significant association between T2DM and RA, we could not further evaluate the potential influence of DPP4i on this association. Secondly, the meta‐analysis included only 12 studies, leading to a relatively small sample size, which might limit the generalizability and robustness of the findings. Although no significant heterogeneity or publication bias was detected, the limited number of studies might have resulted in insufficient statistical power, leaving the possibility of residual bias. Thirdly, this study evaluated the potential impact of DPP4i through its genetic proxies in MR analysis, which is based on genetically predicted DPP4i effects rather than actual drug use data. This limitation suggests that the findings may not fully reflect the real‐world effects of clinical DPP4i use. Future studies using real‐world data or RCTs are needed to further verify the role of DPP4i in RA development. Fourthly, due to the limitations of the available data, we were unable to perform a more comprehensive analysis based on different types of DPP4i and their duration of use. Nevertheless, variations in the type of DPP4i and treatment duration may exert distinct effects on RA, which could potentially influence our study findings. Fifthly, the study relied primarily on GWAS data to investigate the immune regulatory mechanisms of DPP4i. Although we identified potential mediatory effects of specific immune cells and inflammatory cytokines, further cellular and animal model studies are required to directly validate the immunomodulatory effects of DPP4i and clarify its precise role in RA pathogenesis.

## 8. Conclusion

This study revealed a significant association between T2DM and RA, indicating that T2DM may be a potential risk factor for RA. Moreover, DPP4i may reduce the risk of RA through their anti‐inflammatory and immunomodulatory effects. These findings provide new insights into RA prevention and treatment. However, further clinical studies and mechanistic investigations are required to confirm these findings.

## Author Contributions

Lirong Zhang: conceptualization, writing—original draft, writing—review and editing, data curation, formal analysis, methodology, software, and validation. Lan Lin: conceptualization, writing—review and editing, data curation, formal analysis, methodology, software, and validation. Jingting Wang: writing—review and editing, methodology, and project administration. Yixiao Zhu: writing—review and editing, methodology, and project administration. Jiaqin Cai: writing—review and editing. Hong Sun: conceptualization, funding acquisition, supervision, and validation. Xiaoxia Wei: conceptualization, funding acquisition, supervision, and validation. Lirong Zhang and Lan Lin should be regarded as joint first authors.

## Funding

This study was supported by the Natural Science Foundation of Fujian Province (10.13039/501100003392) (2021J01397); Fujian Provincial Health Technology Project (10.13039/501100017686) (2022GGA010); Fujian Provincial Joint Funding Project of Scientific and Technological Innovation (2023Y9347); Wu Jieping Medical Foundation (10.13039/100007452) ([320.6750.2025‐06‐272]).

## Ethics Statement

This study did not involve the collection of individual patient data. Publicly available drug data were used. Therefore, ethics committee approval was not required.

## Conflicts of Interest

The authors declare no conflicts of interest.

## Supporting information


**Supporting Information** Additional supporting information can be found online in the Supporting Information section. File S1: This contains the STROBE checklist. This study was reported in accordance with the STROBE Statement. The completed STROBE checklist is provided in the supporting information to ensure transparent and complete reporting of the observational components of this study. Table S1: Overview of epidemiological studies investigating the associations between T2DM or DPP4i and RA. Table S2: The definitions of relevant variables from the NHANES database used in this study. Table S3: The search strategy for the meta‐analysis conducted in this study. Table S4: Inclusion and exclusion criteria for references. Table S5: Detailed information for genome‐wide association study (GWAS) statistics used in the present study. Table S6 Baseline characteristics of RA group versus the non‐RA arthritis group. Table S7 Description of included studies. Table S8: NOS for quality assessment of included cohort studies. Table S9: The F‐statistics of the IVs for DPP4i used in this study. Table S10: The results of MR‐Egger and weighted median methods evaluating the association between DPP4i and RA. Table S11: The sensitivity analysis results of the MR study. Table S12: The beta and standard error (Se) values in the MR process for inflammatory factors and immune cells that have a causal association with DPP4i and RA. Figure S1: Flowchart of the selection and screening process for NHANES eligible participants from 1999 to 2023. Figure S2: Flow diagram of the literature search and selection criteria. Figure S3: Bias analysis of the included RCTs literature. Figure S4: Evaluation of the quality of the included RCTs literature. Figure S5: The funnel plot of included studies. Figure S6: The result of sensitivity analysis. Figure S7: Sensitivity analysis results of MR study.

## Data Availability

The relevant original source documents are cited in full in the reference section and supporting information. The raw data can be shared with others on reasonable request via email to the corresponding author of this manuscript.
