# Supplementary material for: Assessing the Association Between T2DM, Rheumatoid Arthritis, and Dipeptidyl Peptidase‐4 Inhibitors: Insights From Epidemiology, Meta‐Analysis, and Mendelian Randomization Study
Source: J Diabetes Res. 2026 Mar 11;2026:9011744. doi: 10.1155/jdr/9011744 (PMC12976818; doi:10.1155/jdr/9011744)
Supplement: Supplementary file 1 — Supporting Information Additional supporting information can be found online in the Supporting Information section. File S1: This contains the STROBE checklist. This study was reported in accordance with the STROBE Statement. The completed STROBE checklist is provided in the supporting information to ensure transparent and complete reporting of the observational components of this study. Table S1: Overview of epidemiological studies investigating the associations between T2DM or DPP4i and RA. Table S2: The definitions of relevant variables from the NHANES database used in this study. Table S3: The search strategy for the meta‐analysis conducted in this study. Table S4: Inclusion and exclusion criteria for references. Table S5: Detailed information for genome‐wide association study (GWAS) statistics used in the present study. Table S6 Baseline characteristics of RA group versus the non‐RA arthritis group. Table S7 Description of included studies. Table S8: NOS for quality assessment of included cohort studies. Table S9: The F‐statistics of the IVs for DPP4i used in this study. Table S10: The results of MR‐Egger and weighted median methods evaluating the association between DPP4i and RA. Table S11: The sensitivity analysis results of the MR study. Table S12: The beta and standard error (Se) values in the MR process for inflammatory factors and immune cells that have a causal association with DPP4i and RA. Figure S1: Flowchart of the selection and screening process for NHANES eligible participants from 1999 to 2023. Figure S2: Flow diagram of the literature search and selection criteria. Figure S3: Bias analysis of the included RCTs literature. Figure S4: Evaluation of the quality of the included RCTs literature. Figure S5: The funnel plot of included studies. Figure S6: The result of sensitivity analysis. Figure S7: Sensitivity analysis results of MR study. [file JDR-2026-9011744-s001.docx]

**STROBE-MR checklist of recommended items to address in reports of Mendelian randomization studies**^1^ ^2^

| **Item No.** | **Section** | **Checklist item** | Page No. | Relevant text from manuscript |
| --- | --- | --- | --- | --- |
| 1 | **TITLE and ABSTRACT** | Indicate Mendelian randomization (MR) as the study’s design in the title and/or the abstract if that is a main purpose of the study | 1-3 | TITLE: Assessing the association between T2DM, rheumatoid arthritis, and dipeptidyl peptidase-4 inhibitors: insights from epidemiology, meta-analysis, and mendelian randomization study  Abstract:  Objective: This study explores the association between type 2 diabetes (T2DM), dipeptidyl peptidase-4 inhibitors (DPP4i), and rheumatoid arthritis (RA).  Methods: We conducted a comprehensive analysis using data from the National Health and Nutrition Examination Survey (NHANES), existing studies, and genome-wide association studies (GWAS). Weighted logistic regression was employed to investigate the association between T2DM and RA. A meta-analysis was performed to examine the relationship between DPP4i use and the risk of RA. Additionally, a drug target-mediation mendelian randomization (MR) study was conducted to evaluate the causal relationship between DPP4i and RA, as well as potential pathway mechanisms.  Results: The NHANES analysis revealed T2DM was associated with RA. (OR = 1.31). The meta-analysis, which included 12 studies, indicated a reduced risk of RA among DPP4i users (RR = 0.63). MR analysis demonstrated that DPP4i use was associated with a decreased risk of RA (OR = 0.84). Mediation MR analysis suggested that DPP4i might influence RA development through immune factors such as CD14+CD16+ monocytes, CXCL11, and IL-2 receptors.  Conclusions: This study confirmed a significant association between T2DM and RA and further revealed that DPP4i might reduce RA risk through inflammation and immune modulation. Further studies are needed to confirm these findings. |
|  | **INTRODUCTION** |  |  |  |
| 2 | **Background** | Explain the scientific background and rationale for the reported study. What is the exposure? Is a potential causal relationship between exposure and outcome plausible? Justify why MR is a helpful method to address the study question | 3-7 | Rheumatoid arthritis (RA) is a chronic systemic autoimmune disease characterized by synovial inflammation, joint destruction, and dysregulated systemic immune responses. (1) The etiology of RA remains incompletely understood; however, genetic predisposition, environmental factors, and aberrant activation of the immune system are known to play critical roles in its pathogenesis.(2) In recent years, there has been an increasing emphasis on the role of metabolic disorders in the development of RA, specifically focusing on type 2 diabetes mellitus (T2DM).(3) Several epidemiological studies have revealed the potential positive association between T2DM and RA. (4-6) This indicated the possibility that the two conditions may involve shared inflammatory mechanisms, including insulin resistance, chronic low-grade inflammation, and immune cell dysfunction.(7) However, a cohort study conducted by Jin et al. reported a negative association between T2DM and RA.(8)  Currently, a wide range of therapeutic options are available for the treatment of T2DM, dipeptidyl peptidase-4 inhibitor (DPP4i) have garnered particular attention due to their potential anti-inflammatory and immunomodulatory effects.(9) DPP4, also known as CD26, is a serine protease that is widely expressed in various tissues and plays a crucial role in the immune system. It regulates T cell activation, cytokine secretion, and immune cell infiltration.(10) Previous review articles have summarized evidence suggesting that DPP4i not only regulated blood glucose by inhibiting DPP4 activity but might also influence the development and progression of inflammatory diseases through their immunomodulatory effects.(11) On this basis, preliminary studies have explored the potential association between DPP4i and RA, yielding heterogeneous findings. (12-16) Several observational studies indicated that the use of DPP4i may be associated with a reduced risk of RA. (12, 15, 16) For example, cohort studies reported that T2DM patients treated with DPP4i had a lower incidence of RA compared to those treated with other antidiabetic medications, indicating a possible immunomodulatory role of DPP4i.(12) In contrast, other observational study have failed to identify a significant association between DPP4i use and RA risk,(13) and a limited number of case reports have described the development of RA following DPP4i exposure.(14) The results of the meta-analyses also showed significant differences. A meta-analysis including four cohort studies reported that the use of DPP4i was associated with a reduced risk of RA (RR = 0.72, 95% CI: 0.54–0.96); though heterogeneity was high in this study (I2=75%).(17) A more recent meta-analysis incorporating both cohort studies and randomized controlled trials (RCTs) reported contradictory findings, indicating that DPP4i do not significantly affect the risk of RA (RR = 0.96, 95% CI: 0.69–1.32).(18) Notably, this study did not strictly distinguish between different types of arthritis, osteoarthritis and RA. An overview of representative epidemiological studies exploring the associations between T2DM or DPP4i and RA is presented in the Supplementary Table 1.  Furthermore, the mechanism by which DPP4i influence RA remains unclear. The development of RA involves complex immune regulatory processes, including interactions among various immune cells, such as T cells, B cells, and monocyte-macrophages, as well as inflammatory cytokines, including tumor necrosis factor-alpha (TNF-α), interleukin-6 (IL-6), and IL-17.(19-22) Among these pathways, T cell–mediated immune responses play a central role in RA pathogenesis, and interleukin-2 (IL-2) signaling through IL receptor (IL-2R) is critical for regulating T cell activation, proliferation, and immune tolerance.(23, 24) DPP4i may influence the development and progression of RA by modulating the levels of immune cells and inflammatory cytokines.(25-29) However, current research on whether DPP4i can reduce RA risk through immune-mediated mechanisms remains limited, and systematic studies focusing on specific immune pathways and molecular mechanisms are still lacking.  Current research on the association between T2DM, DPP4i, and RA remains conflicting, with most studies relying on retrospective data, which are limited by confounding factors, making it difficult to establish a causal association between DPP4i and RA.(12-17) Therefore, larger-scale studies and more rigorous methodologies are needed to further investigate the association and underlying mechanisms among T2DM, DPP4i, and RA. The National Health and Nutrition Examination Survey (NHANES), conducted by the National Center for Health Statistics, is a nationally representative, ongoing survey that collects extensive health and nutrition data from thousands of participants annually, providing essential support for comprehensive epidemiological research and generalizable findings. (30) Meta-analysis is a method of quantitatively synthesizing existing studies to derive conclusions that are both comprehensive and reliable.(31, 32) The timely updating of results enhances their reliability and more effectively addresses clinical controversies. Mendelian randomization (MR) studies utilize genetic variants as instrumental variables (IVs), thereby effectively addressing confounding factors and the limitations of causal inference in observational studies. To some extent, MR can approximate the causal inference strength of RCTs.(33) Drug-target MR can be used to investigate the causal association between a specific drug and a disease, providing valuable insights for drug development and personalized treatment.(34) Mediation MR is used to assess potential mediators in a causal pathway, exploring how genetic variants influence outcomes through intermediary variables. This approach helps to identify the specific mechanisms underlying drug effects.(35)  Therefore, we integrated NHANES database analysis, meta-analysis, and drug target-mediated MR studies to provide more reliable evidence on the association between T2DM, DPP4i, and RA from multiple perspectives, with a particular focus on the role of DPP4i in the progression of RA. |
| 3 | **Objectives** | State specific objectives clearly, including pre-specified causal hypotheses (if any). State that MR is a method that, under specific assumptions, intends to estimate causal effects | 2 | Therefore, we integrated NHANES database analysis, meta-analysis, and drug target-mediated MR studies to provide more reliable evidence on the association between T2DM, DPP4i, and RA from multiple perspectives, with a particular focus on the role of DPP4i in the progression of RA. |
|  | **METHODS** |  |  |  |
| 4 | **Study design and data sources** | Present key elements of the study design early in the article. Consider including a table listing sources of data for all phases of the study. For each data source contributing to the analysis, describe the following: | 9 | Supplementary Table 5 |
|  | a) | Setting: Describe the study design and the underlying population, if possible. Describe the setting, locations, and relevant dates, including periods of recruitment, exposure, follow-up, and data collection, when available. | 9-10 | The target of DPP4i is DPP4.(36) To minimize population stratification bias, this study was conducted in a European-ancestry population. The data used for the MR analysis are summarized in Supplementary Table 5.  The selection of IVs followed three fundamental principles: the selected single nucleotide polymorphisms(SNPs) should be strongly associated with the exposure, influence the outcome only through the exposure, and remain independent of potential confounders.(37) To ensure compliance with these principles, we extracted cis-acting SNPs within a 500 kb region surrounding the DPP4 gene from the eQTL database, applying thresholds of P < 5×10⁻⁸, minor allele frequency (MAF) >1%, and false discovery rate (FDR) <0.05. To minimize linkage disequilibrium (LD) bias, independent SNPs were selected using LD clumping (r² < 0.3, 500kb). Additionally, the association between each DPP4 variant and glycated hemoglobin (HbA1c) levels was assessed, and SNPs with an F-statistic <10 was excluded to remove weak instrument bias. We also conducted a colocalization analysis between DPP4 and HbA1c, using a posterior probability threshold of >80% as evidence of colocalization. The final IVs obtained through this process were used as proxies for DPP4i.  This MR study consisted of two main components. In the first step, a two-sample MR analysis was conducted to evaluate the causal association of DPP4i on RA. In the second step, mediation MR was used to evaluate the mediating role of 91 inflammatory factors and 731 immune cells in the DPP4 inhibitor and RA pathways. In the mediation analysis, we first identified immune cells and inflammatory cytokines that had a causal association on RA, then further evaluated those that were also causally associated with DPP4i. The product of coefficients method was used to estimate the mediation effect. In the MR analysis, the inverse-variance weighted (IVW) method was the primary analysis method, as it assumes that more than 50% of the IVs are valid. The MR-Egger regression and weighted median were used as supplementary methods. MR results were reported as odds ratio (OR) with corresponding 95% CI. Sensitivity analyses included Cochran’s Q test, MR-Egger-intercept test, MR-PRESSO, and leave-one-out analysis to assess heterogeneity, horizontal pleiotropy, and robustness. To visualize the results, scatter plots, forest plots, leave-one-out plots, and funnel plots were generated. All statistical analyses were conducted using R software (Version 4.3.1). |
|  | b) | Participants: Give the eligibility criteria, and the sources and methods of selection of participants. Report the sample size, and whether any power or sample size calculations were carried out prior to the main analysis | 9-10 | The target of DPP4i is DPP4.(36) To minimize population stratification bias, this study was conducted in a European-ancestry population. The data used for the MR analysis are summarized in Supplementary Table 5.  The selection of IVs followed three fundamental principles: the selected single nucleotide polymorphisms(SNPs) should be strongly associated with the exposure, influence the outcome only through the exposure, and remain independent of potential confounders.(37) To ensure compliance with these principles, we extracted cis-acting SNPs within a 500 kb region surrounding the DPP4 gene from the eQTL database, applying thresholds of P < 5×10⁻⁸, minor allele frequency (MAF) >1%, and false discovery rate (FDR) <0.05. To minimize linkage disequilibrium (LD) bias, independent SNPs were selected using LD clumping (r² < 0.3, 500kb). Additionally, the association between each DPP4 variant and glycated hemoglobin (HbA1c) levels was assessed, and SNPs with an F-statistic <10 was excluded to remove weak instrument bias. We also conducted a colocalization analysis between DPP4 and HbA1c, using a posterior probability threshold of >80% as evidence of colocalization. The final IVs obtained through this process were used as proxies for DPP4i.  This MR study consisted of two main components. In the first step, a two-sample MR analysis was conducted to evaluate the causal association of DPP4i on RA. In the second step, mediation MR was used to evaluate the mediating role of 91 inflammatory factors and 731 immune cells in the DPP4 inhibitor and RA pathways. In the mediation analysis, we first identified immune cells and inflammatory cytokines that had a causal association on RA, then further evaluated those that were also causally associated with DPP4i. The product of coefficients method was used to estimate the mediation effect. In the MR analysis, the inverse-variance weighted (IVW) method was the primary analysis method, as it assumes that more than 50% of the IVs are valid. The MR-Egger regression and weighted median were used as supplementary methods. MR results were reported as odds ratio (OR) with corresponding 95% CI. Sensitivity analyses included Cochran’s Q test, MR-Egger-intercept test, MR-PRESSO, and leave-one-out analysis to assess heterogeneity, horizontal pleiotropy, and robustness. To visualize the results, scatter plots, forest plots, leave-one-out plots, and funnel plots were generated. All statistical analyses were conducted using R software (Version 4.3.1). |
|  | c) | Describe measurement, quality control and selection of genetic variants | 9 | The target of DPP4i is DPP4.(36) To minimize population stratification bias, this study was conducted in a European-ancestry population. The data used for the MR analysis are summarized in Supplementary Table 5. |
|  | d) | For each exposure, outcome, and other relevant variables, describe methods of assessment and diagnostic criteria for diseases | 9 | Supplementary Table 5 |
|  | e) | Provide details of ethics committee approval and participant informed consent, if relevant | 7 | In this study, we utilized publicly available data that had received appropriate ethical approvals. As the study was based on aggregated, de-identified genetic data, institutional review board approval and informed consent were not required. This study adhered to the Strengthening the Reporting of Observational Studies in Epidemiology using Mendelian Randomization (STROBE-MR) guidelines for transparent and standardized reporting. |
| 5 | **Assumptions** | Explicitly state the three core IV assumptions for the main analysis (relevance, independence and exclusion restriction) as well assumptions for any additional or sensitivity analysis | 9-10 | The selection of IVs followed three fundamental principles: the selected single nucleotide polymorphisms(SNPs) should be strongly associated with the exposure, influence the outcome only through the exposure, and remain independent of potential confounders.(37) To ensure compliance with these principles, we extracted cis-acting SNPs within a 500 kb region surrounding the DPP4 gene from the eQTL database, applying thresholds of P < 5×10⁻⁸, minor allele frequency (MAF) >1%, and false discovery rate (FDR) <0.05. To minimize linkage disequilibrium (LD) bias, independent SNPs were selected using LD clumping (r² < 0.3, 500kb). Additionally, the association between each DPP4 variant and glycated hemoglobin (HbA1c) levels was assessed, and SNPs with an F-statistic <10 was excluded to remove weak instrument bias. We also conducted a colocalization analysis between DPP4 and HbA1c, using a posterior probability threshold of >80% as evidence of colocalization. The final IVs obtained through this process were used as proxies for DPP4i.  This MR study consisted of two main components. In the first step, a two-sample MR analysis was conducted to evaluate the causal association of DPP4i on RA. In the second step, mediation MR was used to evaluate the mediating role of 91 inflammatory factors and 731 immune cells in the DPP4 inhibitor and RA pathways. In the mediation analysis, we first identified immune cells and inflammatory cytokines that had a causal association on RA, then further evaluated those that were also causally associated with DPP4i. The product of coefficients method was used to estimate the mediation effect. In the MR analysis, the inverse-variance weighted (IVW) method was the primary analysis method, as it assumes that more than 50% of the IVs are valid. The MR-Egger regression and weighted median were used as supplementary methods. MR results were reported as odds ratio (OR) with corresponding 95% CI. Sensitivity analyses included Cochran’s Q test, MR-Egger-intercept test, MR-PRESSO, and leave-one-out analysis to assess heterogeneity, horizontal pleiotropy, and robustness. To visualize the results, scatter plots, forest plots, leave-one-out plots, and funnel plots were generated. All statistical analyses were conducted using R software (Version 4.3.1). |
| 6 | **Statistical methods: main analysis** | Describe statistical methods and statistics used |  |  |
|  | a) | Describe how quantitative variables were handled in the analyses (i.e., scale, units, model) | 10 | This MR study consisted of two main components. In the first step, a two-sample MR analysis was conducted to evaluate the causal association of DPP4i on RA. In the second step, mediation MR was used to evaluate the mediating role of 91 inflammatory factors and 731 immune cells in the DPP4 inhibitor and RA pathways. In the mediation analysis, we first identified immune cells and inflammatory cytokines that had a causal association on RA, then further evaluated those that were also causally associated with DPP4i. The product of coefficients method was used to estimate the mediation effect. In the MR analysis, the inverse-variance weighted (IVW) method was the primary analysis method, as it assumes that more than 50% of the IVs are valid. The MR-Egger regression and weighted median were used as supplementary methods. MR results were reported as odds ratio (OR) with corresponding 95% CI. Sensitivity analyses included Cochran’s Q test, MR-Egger-intercept test, MR-PRESSO, and leave-one-out analysis to assess heterogeneity, horizontal pleiotropy, and robustness. To visualize the results, scatter plots, forest plots, leave-one-out plots, and funnel plots were generated. All statistical analyses were conducted using R software (Version 4.3.1). |
|  | b) | Describe how genetic variants were handled in the analyses and, if applicable, how their weights were selected | 10 | The selection of IVs followed three fundamental principles: the selected single nucleotide polymorphisms(SNPs) should be strongly associated with the exposure, influence the outcome only through the exposure, and remain independent of potential confounders.(37) To ensure compliance with these principles, we extracted cis-acting SNPs within a 500 kb region surrounding the DPP4 gene from the eQTL database, applying thresholds of P < 5×10⁻⁸, minor allele frequency (MAF) >1%, and false discovery rate (FDR) <0.05. To minimize linkage disequilibrium (LD) bias, independent SNPs were selected using LD clumping (r² < 0.3, 500kb). Additionally, the association between each DPP4 variant and glycated hemoglobin (HbA1c) levels was assessed, and SNPs with an F-statistic <10 was excluded to remove weak instrument bias. We also conducted a colocalization analysis between DPP4 and HbA1c, using a posterior probability threshold of >80% as evidence of colocalization. The final IVs obtained through this process were used as proxies for DPP4i. |
|  | c) | Describe the MR estimator (e.g. two-stage least squares, Wald ratio) and related statistics. Detail the included covariates and, in case of two-sample MR, whether the same covariate set was used for adjustment in the two samples | 10 | The product of coefficients method was used to estimate the mediation effect. In the MR analysis, the inverse-variance weighted (IVW) method was the primary analysis method, as it assumes that more than 50% of the IVs are valid. The MR-Egger regression and weighted median were used as supplementary methods. MR results were reported as odds ratio (OR) with corresponding 95% CI. |
|  | d) | Explain how missing data were addressed | NA | Used GWAS summary statistics |
|  | e) | If applicable, indicate how multiple testing was addressed | NA |  |
| 7 | **Assessment of assumptions** | Describe any methods or prior knowledge used to assess the assumptions or justify their validity | 10 | The product of coefficients method was used to estimate the mediation effect. In the MR analysis, the inverse-variance weighted (IVW) method was the primary analysis method, as it assumes that more than 50% of the IVs are valid. The MR-Egger regression and weighted median were used as supplementary methods. MR results were reported as odds ratio (OR) with corresponding 95% CI. Sensitivity analyses included Cochran’s Q test, MR-Egger-intercept test, MR-PRESSO, and leave-one-out analysis to assess heterogeneity, horizontal pleiotropy, and robustness. To visualize the results, scatter plots, forest plots, leave-one-out plots, and funnel plots were generated. All statistical analyses were conducted using R software (Version 4.3.1). |
| 8 | **Sensitivity analyses and additional analyses** | Describe any sensitivity analyses or additional analyses performed (e.g. comparison of effect estimates from different approaches, independent replication, bias analytic techniques, validation of instruments, simulations) | 10 | The product of coefficients method was used to estimate the mediation effect. In the MR analysis, the inverse-variance weighted (IVW) method was the primary analysis method, as it assumes that more than 50% of the IVs are valid. The MR-Egger regression and weighted median were used as supplementary methods. MR results were reported as odds ratio (OR) with corresponding 95% CI. Sensitivity analyses included Cochran’s Q test, MR-Egger-intercept test, MR-PRESSO, and leave-one-out analysis to assess heterogeneity, horizontal pleiotropy, and robustness. To visualize the results, scatter plots, forest plots, leave-one-out plots, and funnel plots were generated. All statistical analyses were conducted using R software (Version 4.3.1). |
| 9 | **Software and pre-registration** |  |  |  |
|  | a) | Name statistical software and package(s), including version and settings used | 10 | R software (Version 4.3.1). |
|  | b) | State whether the study protocol and details were pre-registered (as well as when and where) | NA | NA |
|  | **RESULTS** |  |  |  |
| 10 | **Descriptive data** |  |  |  |
|  | a) | Report the numbers of individuals at each stage of included studies and reasons for exclusion. Consider use of a flow diagram | NA | Used publicly available GWAS summary statistics |
|  | b) | Report summary statistics for phenotypic exposure(s), outcome(s), and other relevant variables (e.g. means, SDs, proportions) | 9 | Supplementary Table 5 |
|  | c) | If the data sources include meta-analyses of previous studies, provide the assessments of heterogeneity across these studies | NA | NA |
|  | d) | For two-sample MR:  i.  Provide justification of the similarity of the genetic variant-exposure associations between the exposure and outcome samples  ii.  Provide information on the number of individuals who overlap between the exposure and outcome studies | 9 | Supplementary Table 5 |
| 11 | **Main results** |  |  |  |
|  | a) | Report the associations between genetic variant and exposure, and between genetic variant and outcome, preferably on an interpretable scale | 17-18 | Based on the IVs selection criteria, 14 SNPs were identified as genetic proxies for DPP4i effects. All SNPs had F-statistics greater than 10, eliminating the influence of weak instruments. (Supplementary Table 9) The IVW model analysis demonstrated a causal association between DPP4i and RA, indicating that for each SD increase in DPP4i, the risk of RA decreased by 16% (OR = 0.84, 95% CI: 0.76–0.93). (Figure 6) The MR-Egger and weighted median yielded consistent results. (Supplementary Table 10) Sensitivity analyses detected no evidence of heterogeneity or horizontal pleiotropy (P values of Cochran Q test, MR-PRESSO and MR-Egger intercept test were all >0.05). (Supplementary Table 11) Leave-one-out analysis revealed that no single SNP had an obvious influence on the causal inference. (Supplementary Figure 7) |
|  | b) | Report MR estimates of the relationship between exposure and outcome, and the measures of uncertainty from the MR analysis, on an interpretable scale, such as odds ratio or relative risk per SD difference | 18-19 | We estimated the effects of 91 inflammatory cytokines and 731 immune cell types on RA and then identified 15 inflammatory cytokines and 11 immune cell types that exhibited a causal association with RA. Further analysis of the association between DPP4i and these inflammatory cytokines and immune cells revealed that DPP4i was causally associated with 8 inflammatory cytokines and 11 immune cell types. (Supplementary Table 12) Mediation effect analysis and mediation proportion calculations were conducted for the identified inflammatory cytokines and immune cells. The results indicated that 2 inflammatory cytokines and 4 immune cell types acted as mediators in the association between DPP4i and RA. (Figure 5) As shown in Table 2, DPP4i exerted an indirect effect on RA risk through CD14⁺ CD16⁺ monocyte AC, CD14⁺ CD16⁺ monocyte %monocyte, CD11b on CD14⁺ monocyte, CD62L⁻ HLA-DR⁺⁺ monocyte AC, C-X-C motif chemokine 11 (CXCL11) levels, and interleukin-2 receptor subunit beta (IL-2Rβ) levels. The proportions of the total effect mediated by these factors were 2%, 15%, 14%, 13%, 11%, and 10%, respectively. (Table 2) |
|  | c) | If relevant, consider translating estimates of relative risk into absolute risk for a meaningful time period | NA | NA |
|  | d) | Consider plots to visualize results (e.g. forest plot, scatterplot of associations between genetic variants and outcome versus between genetic variants and exposure) | Figure 6 and Supplementary Figure 7 | Figure 6 and Supplementary Figure 7 |
| 12 | **Assessment of assumptions** |  |  |  |
|  | a) | Report the assessment of the validity of the assumptions | Table 2,  Supplemental file Table S1-4 | This study used publicly available GWAS summary statistics which limits the assessment for testing the assumptions .The F statistic are in Supplemental file Table S1-4. |
|  | b) | Report any additional statistics (e.g., assessments of heterogeneity across genetic variants, such as I2, Q statistic or E-value) | 17 | Based on the IVs selection criteria, 14 SNPs were identified as genetic proxies for DPP4i effects. All SNPs had F-statistics greater than 10, eliminating the influence of weak instruments. (Supplementary Table 9) |
| 13 | **Sensitivity analyses and additional analyses** |  |  |  |
|  | a) | Report any sensitivity analyses to assess the robustness of the main results to violations of the assumptions | 17-18 | The MR-Egger and weighted median yielded consistent results. (Supplementary Table 10) Sensitivity analyses detected no evidence of heterogeneity or horizontal pleiotropy (P values of Cochran Q test, MR-PRESSO and MR-Egger intercept test were all >0.05). (Supplementary Table 11) Leave-one-out analysis revealed that no single SNP had an obvious influence on the causal inference. (Supplementary Figure 7) |
|  | b) | Report results from other sensitivity analyses or additional analyses | NA | NA |
|  | c) | Report any assessment of direction of causal relationship (e.g., bidirectional MR) | 17 | The IVW model analysis demonstrated a causal association between DPP4i and RA, indicating that for each SD increase in DPP4i, the risk of RA decreased by 16% (OR = 0.84, 95% CI: 0.76–0.93). (Figure 6) |
|  | d) | When relevant, report and compare with estimates from non-MR analyses | NA | NA |
|  | e) | Consider additional plots to visualize results (e.g., leave-one-out analyses) | Supplementary Figure 7 | Supplementary Figure 7 |
|  | **DISCUSSION** |  |  |  |
| 14 | **Key results** | Summarize key results with reference to study objectives | 20 | Our findings provided compelling evidence demonstrating that T2DM may be a risk factor for RA, and the protective effect of DPP4i on RA may be mediated through immune pathways. |
| 15 | **Limitations** | Discuss limitations of the study, taking into account the validity of the IV assumptions, other sources of potential bias, and imprecision. Discuss both direction and magnitude of any potential bias and any efforts to address them | 24-25 | Thirdly, this study evaluated the potential impact of DPP4i through its genetic proxies in MR analysis, which is based on genetically predicted DPP4i effects rather than actual drug use data. This limitation suggested that the findings may not fully reflect the real-world effects of clinical DPP4i use.  Nevertheless, variations in the type of DPP4i and treatment duration may exert distinct effects on RA, which could potentially influence our study findings. Fifthly, the study relied primarily on GWAS data to investigate the immune regulatory mechanisms of DPP4i. While we identified potential mediatory effects of specific immune cells and inflammatory cytokines, further cellular and animal model studies are required to directly validate the immunomodulatory effects of DPP4i and clarify its precise role in RA pathogenesis. |
| 16 | **Interpretation** |  |  |  |
|  | a) | Meaning: Give a cautious overall interpretation of results in the context of their limitations and in comparison with other studies | 24-25 | Thirdly, this study evaluated the potential impact of DPP4i through its genetic proxies in MR analysis, which is based on genetically predicted DPP4i effects rather than actual drug use data. This limitation suggested that the findings may not fully reflect the real-world effects of clinical DPP4i use.  Nevertheless, variations in the type of DPP4i and treatment duration may exert distinct effects on RA, which could potentially influence our study findings. Fifthly, the study relied primarily on GWAS data to investigate the immune regulatory mechanisms of DPP4i. While we identified potential mediatory effects of specific immune cells and inflammatory cytokines, further cellular and animal model studies are required to directly validate the immunomodulatory effects of DPP4i and clarify its precise role in RA pathogenesis. |
|  | b) | Mechanism: Discuss underlying biological mechanisms that could drive a potential causal relationship between the investigated exposure and the outcome, and whether the gene-environment equivalence assumption is reasonable. Use causal language carefully, clarifying that IV estimates may provide causal effects only under certain assumptions | 22-23 | Although multiple studies have investigated the anti-inflammatory and immunomodulatory effects of DPP4i, there have no experimental studies have specifically examined the mechanistic pathways linking DPP4i to RA. Therefore, we conducted a mediation MR analysis, which revealed that DPP4i might reduce RA risk by modulating specific immune cells and inflammatory cytokines. Studies have shown that CD14⁺CD16⁺ monocytes are highly enriched in RA synovial tissue and can promote the release of pro-inflammatory cytokines, such as TNF-α and IL-1β, thereby exacerbating RA-associated inflammatory responses.(53, 54) This study found that DPP4i can modulate the expression of CD14⁺CD16⁺ monocytes, suggesting that it may influence RA progression by regulating the infiltration of pro-inflammatory monocytes. Meanwhile, CXCL11, a chemokine significantly elevated in the serum of RA patients, plays a key role in promoting T cell and monocyte recruitment, thereby exacerbating synovial inflammation.(55-57) This study suggested that DPP4i might reduce RA risk by inhibiting CXCL11 expression, thereby limiting the infiltration of inflammatory cells and mitigating synovial inflammation. The IL-2/IL-2R plays a crucial role in immune regulation in RA, abnormal IL-2R expression may lead to excessive T-cell activation, thereby exacerbating RA-associated immunopathology.(58) This study further demonstrated that DPP4i might modulate IL-2R signaling, potentially contributing to the restoration of immune balance in RA. These research results indicated that DPP4i might play a role in RA immune regulation, providing a new research direction for the potential therapeutic applications of DPP4i in autoimmune diseases. |
|  | c) | Clinical relevance: Discuss whether the results have clinical or public policy relevance, and to what extent they inform effect sizes of possible interventions | 23 | These research results indicated that DPP4i might play a role in RA immune regulation, providing a new research direction for the potential therapeutic applications of DPP4i in autoimmune diseases. |
| 17 | **Generalizability** | Discuss the generalizability of the study results (a) to other populations, (b) across other exposure periods/timings, and (c) across other levels of exposure | 24-25 | Thirdly, this study evaluated the potential impact of DPP4i through its genetic proxies in MR analysis, which is based on genetically predicted DPP4i effects rather than actual drug use data. This limitation suggested that the findings may not fully reflect the real-world effects of clinical DPP4i use.  Nevertheless, variations in the type of DPP4i and treatment duration may exert distinct effects on RA, which could potentially influence our study findings. Fifthly, the study relied primarily on GWAS data to investigate the immune regulatory mechanisms of DPP4i. While we identified potential mediatory effects of specific immune cells and inflammatory cytokines, further cellular and animal model studies are required to directly validate the immunomodulatory effects of DPP4i and clarify its precise role in RA pathogenesis. |
|  | **OTHER INFORMATION** |  |  |  |
| 18 | **Funding** | Describe sources of funding and the role of funders in the present study and, if applicable, sources of funding for the databases and original study or studies on which the present study is based | 25 | This work was supported by the Natural Science Foundation of Fujian, China [grant number 2021J01397]; the Fujian provincial health technology project [grant number 2022GGA010]; the Fujian provincial Joint Funding Project of Scientific and Technological Innovation [grant numbers 2023Y9347]. All authors acknowledge that they had access to all study data and are responsible for the decision to submit for publication. |
| 19 | **Data and data sharing** | Provide the data used to perform all analyses or report where and how the data can be accessed, and reference these sources in the article. Provide the statistical code needed to reproduce the results in the article, or report whether the code is publicly accessible and if so, where | NA | Used publicly available GWAS summary statistics |
| 20 | **Conflicts of Interest** | All authors should declare all potential conflicts of interest | 25 | The authors have no relevant financial or non-financial interests to disclose. |
| **Item No.** | **Section** | Checklist item | **Page No.** | Relevant text from manuscript |
| 1 | **TITLE and ABSTRACT** | Indicate Mendelian randomization (MR) as the study’s design in the title and/or the abstract if that is a main purpose of the study | 1-3 | TITLE: Assessing the association between T2DM, rheumatoid arthritis, and dipeptidyl peptidase-4 inhibitors: insights from epidemiology, meta-analysis, and mendelian randomization study  Abstract:  Objective: This study explores the association between type 2 diabetes (T2DM), dipeptidyl peptidase-4 inhibitors (DPP4i), and rheumatoid arthritis (RA).  Methods: We conducted a comprehensive analysis using data from the National Health and Nutrition Examination Survey (NHANES), existing studies, and genome-wide association studies (GWAS). Weighted logistic regression was employed to investigate the association between T2DM and RA. A meta-analysis was performed to examine the relationship between DPP4i use and the risk of RA. Additionally, a drug target-mediation mendelian randomization (MR) study was conducted to evaluate the causal relationship between DPP4i and RA, as well as potential pathway mechanisms.  Results: The NHANES analysis revealed T2DM was associated with RA. (OR = 1.31). The meta-analysis, which included 12 studies, indicated a reduced risk of RA among DPP4i users (RR = 0.63). MR analysis demonstrated that DPP4i use was associated with a decreased risk of RA (OR = 0.84). Mediation MR analysis suggested that DPP4i might influence RA development through immune factors such as CD14+CD16+ monocytes, CXCL11, and IL-2 receptors.  Conclusions: This study confirmed a significant association between T2DM and RA and further revealed that DPP4i might reduce RA risk through inflammation and immune modulation. Further studies are needed to confirm these findings. |
|  | **INTRODUCTION** |  |  |  |
| 2 | **Background** | Explain the scientific background and rationale for the reported study. What is the exposure? Is a potential causal relationship between exposure and outcome plausible? Justify why MR is a helpful method to address the study question | 3-7 | Rheumatoid arthritis (RA) is a chronic systemic autoimmune disease characterized by synovial inflammation, joint destruction, and dysregulated systemic immune responses. (1) The etiology of RA remains incompletely understood; however, genetic predisposition, environmental factors, and aberrant activation of the immune system are known to play critical roles in its pathogenesis.(2) In recent years, there has been an increasing emphasis on the role of metabolic disorders in the development of RA, specifically focusing on type 2 diabetes mellitus (T2DM).(3) Several epidemiological studies have revealed the potential positive association between T2DM and RA. (4-6) This indicated the possibility that the two conditions may involve shared inflammatory mechanisms, including insulin resistance, chronic low-grade inflammation, and immune cell dysfunction.(7) However, a cohort study conducted by Jin et al. reported a negative association between T2DM and RA.(8)  Currently, a wide range of therapeutic options are available for the treatment of T2DM, dipeptidyl peptidase-4 inhibitor (DPP4i) have garnered particular attention due to their potential anti-inflammatory and immunomodulatory effects.(9) DPP4, also known as CD26, is a serine protease that is widely expressed in various tissues and plays a crucial role in the immune system. It regulates T cell activation, cytokine secretion, and immune cell infiltration.(10) Previous review articles have summarized evidence suggesting that DPP4i not only regulated blood glucose by inhibiting DPP4 activity but might also influence the development and progression of inflammatory diseases through their immunomodulatory effects.(11) On this basis, preliminary studies have explored the potential association between DPP4i and RA, yielding heterogeneous findings. (12-16) Several observational studies indicated that the use of DPP4i may be associated with a reduced risk of RA. (12, 15, 16) For example, cohort studies reported that T2DM patients treated with DPP4i had a lower incidence of RA compared to those treated with other antidiabetic medications, indicating a possible immunomodulatory role of DPP4i.(12) In contrast, other observational study have failed to identify a significant association between DPP4i use and RA risk,(13) and a limited number of case reports have described the development of RA following DPP4i exposure.(14) The results of the meta-analyses also showed significant differences. A meta-analysis including four cohort studies reported that the use of DPP4i was associated with a reduced risk of RA (RR = 0.72, 95% CI: 0.54–0.96); though heterogeneity was high in this study (I2=75%).(17) A more recent meta-analysis incorporating both cohort studies and randomized controlled trials (RCTs) reported contradictory findings, indicating that DPP4i do not significantly affect the risk of RA (RR = 0.96, 95% CI: 0.69–1.32).(18) Notably, this study did not strictly distinguish between different types of arthritis, osteoarthritis and RA. An overview of representative epidemiological studies exploring the associations between T2DM or DPP4i and RA is presented in the Supplementary Table 1.  Furthermore, the mechanism by which DPP4i influence RA remains unclear. The development of RA involves complex immune regulatory processes, including interactions among various immune cells, such as T cells, B cells, and monocyte-macrophages, as well as inflammatory cytokines, including tumor necrosis factor-alpha (TNF-α), interleukin-6 (IL-6), and IL-17.(19-22) Among these pathways, T cell–mediated immune responses play a central role in RA pathogenesis, and interleukin-2 (IL-2) signaling through IL receptor (IL-2R) is critical for regulating T cell activation, proliferation, and immune tolerance.(23, 24) DPP4i may influence the development and progression of RA by modulating the levels of immune cells and inflammatory cytokines.(25-29) However, current research on whether DPP4i can reduce RA risk through immune-mediated mechanisms remains limited, and systematic studies focusing on specific immune pathways and molecular mechanisms are still lacking.  Current research on the association between T2DM, DPP4i, and RA remains conflicting, with most studies relying on retrospective data, which are limited by confounding factors, making it difficult to establish a causal association between DPP4i and RA.(12-17) Therefore, larger-scale studies and more rigorous methodologies are needed to further investigate the association and underlying mechanisms among T2DM, DPP4i, and RA. The National Health and Nutrition Examination Survey (NHANES), conducted by the National Center for Health Statistics, is a nationally representative, ongoing survey that collects extensive health and nutrition data from thousands of participants annually, providing essential support for comprehensive epidemiological research and generalizable findings. (30) Meta-analysis is a method of quantitatively synthesizing existing studies to derive conclusions that are both comprehensive and reliable.(31, 32) The timely updating of results enhances their reliability and more effectively addresses clinical controversies. Mendelian randomization (MR) studies utilize genetic variants as instrumental variables (IVs), thereby effectively addressing confounding factors and the limitations of causal inference in observational studies. To some extent, MR can approximate the causal inference strength of RCTs.(33) Drug-target MR can be used to investigate the causal association between a specific drug and a disease, providing valuable insights for drug development and personalized treatment.(34) Mediation MR is used to assess potential mediators in a causal pathway, exploring how genetic variants influence outcomes through intermediary variables. This approach helps to identify the specific mechanisms underlying drug effects.(35)  Therefore, we integrated NHANES database analysis, meta-analysis, and drug target-mediated MR studies to provide more reliable evidence on the association between T2DM, DPP4i, and RA from multiple perspectives, with a particular focus on the role of DPP4i in the progression of RA. |
| 3 | **Objectives** | State specific objectives clearly, including pre-specified causal hypotheses (if any). State that MR is a method that, under specific assumptions, intends to estimate causal effects | 2 | Therefore, we integrated NHANES database analysis, meta-analysis, and drug target-mediated MR studies to provide more reliable evidence on the association between T2DM, DPP4i, and RA from multiple perspectives, with a particular focus on the role of DPP4i in the progression of RA. |
|  | **METHODS** |  |  |  |
| 4 | **Study design and data sources** | Present key elements of the study design early in the article. Consider including a table listing sources of data for all phases of the study. For each data source contributing to the analysis, describe the following: | 9 | Supplementary Table 5 |
|  | a) | Setting: Describe the study design and the underlying population, if possible. Describe the setting, locations, and relevant dates, including periods of recruitment, exposure, follow-up, and data collection, when available. | 9-10 | The target of DPP4i is DPP4.(36) To minimize population stratification bias, this study was conducted in a European-ancestry population. The data used for the MR analysis are summarized in Supplementary Table 5.  The selection of IVs followed three fundamental principles: the selected single nucleotide polymorphisms(SNPs) should be strongly associated with the exposure, influence the outcome only through the exposure, and remain independent of potential confounders.(37) To ensure compliance with these principles, we extracted cis-acting SNPs within a 500 kb region surrounding the DPP4 gene from the eQTL database, applying thresholds of P < 5×10⁻⁸, minor allele frequency (MAF) >1%, and false discovery rate (FDR) <0.05. To minimize linkage disequilibrium (LD) bias, independent SNPs were selected using LD clumping (r² < 0.3, 500kb). Additionally, the association between each DPP4 variant and glycated hemoglobin (HbA1c) levels was assessed, and SNPs with an F-statistic <10 was excluded to remove weak instrument bias. We also conducted a colocalization analysis between DPP4 and HbA1c, using a posterior probability threshold of >80% as evidence of colocalization. The final IVs obtained through this process were used as proxies for DPP4i.  This MR study consisted of two main components. In the first step, a two-sample MR analysis was conducted to evaluate the causal association of DPP4i on RA. In the second step, mediation MR was used to evaluate the mediating role of 91 inflammatory factors and 731 immune cells in the DPP4 inhibitor and RA pathways. In the mediation analysis, we first identified immune cells and inflammatory cytokines that had a causal association on RA, then further evaluated those that were also causally associated with DPP4i. The product of coefficients method was used to estimate the mediation effect. In the MR analysis, the inverse-variance weighted (IVW) method was the primary analysis method, as it assumes that more than 50% of the IVs are valid. The MR-Egger regression and weighted median were used as supplementary methods. MR results were reported as odds ratio (OR) with corresponding 95% CI. Sensitivity analyses included Cochran’s Q test, MR-Egger-intercept test, MR-PRESSO, and leave-one-out analysis to assess heterogeneity, horizontal pleiotropy, and robustness. To visualize the results, scatter plots, forest plots, leave-one-out plots, and funnel plots were generated. All statistical analyses were conducted using R software (Version 4.3.1). |
|  | b) | Participants: Give the eligibility criteria, and the sources and methods of selection of participants. Report the sample size, and whether any power or sample size calculations were carried out prior to the main analysis | 9-10 | The target of DPP4i is DPP4.(36) To minimize population stratification bias, this study was conducted in a European-ancestry population. The data used for the MR analysis are summarized in Supplementary Table 5.  The selection of IVs followed three fundamental principles: the selected single nucleotide polymorphisms(SNPs) should be strongly associated with the exposure, influence the outcome only through the exposure, and remain independent of potential confounders.(37) To ensure compliance with these principles, we extracted cis-acting SNPs within a 500 kb region surrounding the DPP4 gene from the eQTL database, applying thresholds of P < 5×10⁻⁸, minor allele frequency (MAF) >1%, and false discovery rate (FDR) <0.05. To minimize linkage disequilibrium (LD) bias, independent SNPs were selected using LD clumping (r² < 0.3, 500kb). Additionally, the association between each DPP4 variant and glycated hemoglobin (HbA1c) levels was assessed, and SNPs with an F-statistic <10 was excluded to remove weak instrument bias. We also conducted a colocalization analysis between DPP4 and HbA1c, using a posterior probability threshold of >80% as evidence of colocalization. The final IVs obtained through this process were used as proxies for DPP4i.  This MR study consisted of two main components. In the first step, a two-sample MR analysis was conducted to evaluate the causal association of DPP4i on RA. In the second step, mediation MR was used to evaluate the mediating role of 91 inflammatory factors and 731 immune cells in the DPP4 inhibitor and RA pathways. In the mediation analysis, we first identified immune cells and inflammatory cytokines that had a causal association on RA, then further evaluated those that were also causally associated with DPP4i. The product of coefficients method was used to estimate the mediation effect. In the MR analysis, the inverse-variance weighted (IVW) method was the primary analysis method, as it assumes that more than 50% of the IVs are valid. The MR-Egger regression and weighted median were used as supplementary methods. MR results were reported as odds ratio (OR) with corresponding 95% CI. Sensitivity analyses included Cochran’s Q test, MR-Egger-intercept test, MR-PRESSO, and leave-one-out analysis to assess heterogeneity, horizontal pleiotropy, and robustness. To visualize the results, scatter plots, forest plots, leave-one-out plots, and funnel plots were generated. All statistical analyses were conducted using R software (Version 4.3.1). |
|  | c) | Describe measurement, quality control and selection of genetic variants | 9 | The target of DPP4i is DPP4.(36) To minimize population stratification bias, this study was conducted in a European-ancestry population. The data used for the MR analysis are summarized in Supplementary Table 5. |
|  | d) | For each exposure, outcome, and other relevant variables, describe methods of assessment and diagnostic criteria for diseases | 9 | Supplementary Table 5 |
|  | e) | Provide details of ethics committee approval and participant informed consent, if relevant | 7 | In this study, we utilized publicly available data that had received appropriate ethical approvals. As the study was based on aggregated, de-identified genetic data, institutional review board approval and informed consent were not required. This study adhered to the Strengthening the Reporting of Observational Studies in Epidemiology using Mendelian Randomization (STROBE-MR) guidelines for transparent and standardized reporting. |
| 5 | **Assumptions** | Explicitly state the three core IV assumptions for the main analysis (relevance, independence and exclusion restriction) as well assumptions for any additional or sensitivity analysis | 9-10 | The selection of IVs followed three fundamental principles: the selected single nucleotide polymorphisms(SNPs) should be strongly associated with the exposure, influence the outcome only through the exposure, and remain independent of potential confounders.(37) To ensure compliance with these principles, we extracted cis-acting SNPs within a 500 kb region surrounding the DPP4 gene from the eQTL database, applying thresholds of P < 5×10⁻⁸, minor allele frequency (MAF) >1%, and false discovery rate (FDR) <0.05. To minimize linkage disequilibrium (LD) bias, independent SNPs were selected using LD clumping (r² < 0.3, 500kb). Additionally, the association between each DPP4 variant and glycated hemoglobin (HbA1c) levels was assessed, and SNPs with an F-statistic <10 was excluded to remove weak instrument bias. We also conducted a colocalization analysis between DPP4 and HbA1c, using a posterior probability threshold of >80% as evidence of colocalization. The final IVs obtained through this process were used as proxies for DPP4i.  This MR study consisted of two main components. In the first step, a two-sample MR analysis was conducted to evaluate the causal association of DPP4i on RA. In the second step, mediation MR was used to evaluate the mediating role of 91 inflammatory factors and 731 immune cells in the DPP4 inhibitor and RA pathways. In the mediation analysis, we first identified immune cells and inflammatory cytokines that had a causal association on RA, then further evaluated those that were also causally associated with DPP4i. The product of coefficients method was used to estimate the mediation effect. In the MR analysis, the inverse-variance weighted (IVW) method was the primary analysis method, as it assumes that more than 50% of the IVs are valid. The MR-Egger regression and weighted median were used as supplementary methods. MR results were reported as odds ratio (OR) with corresponding 95% CI. Sensitivity analyses included Cochran’s Q test, MR-Egger-intercept test, MR-PRESSO, and leave-one-out analysis to assess heterogeneity, horizontal pleiotropy, and robustness. To visualize the results, scatter plots, forest plots, leave-one-out plots, and funnel plots were generated. All statistical analyses were conducted using R software (Version 4.3.1). |
| 6 | **Statistical methods: main analysis** | Describe statistical methods and statistics used |  |  |
|  | a) | Describe how quantitative variables were handled in the analyses (i.e., scale, units, model) | 10 | This MR study consisted of two main components. In the first step, a two-sample MR analysis was conducted to evaluate the causal association of DPP4i on RA. In the second step, mediation MR was used to evaluate the mediating role of 91 inflammatory factors and 731 immune cells in the DPP4 inhibitor and RA pathways. In the mediation analysis, we first identified immune cells and inflammatory cytokines that had a causal association on RA, then further evaluated those that were also causally associated with DPP4i. The product of coefficients method was used to estimate the mediation effect. In the MR analysis, the inverse-variance weighted (IVW) method was the primary analysis method, as it assumes that more than 50% of the IVs are valid. The MR-Egger regression and weighted median were used as supplementary methods. MR results were reported as odds ratio (OR) with corresponding 95% CI. Sensitivity analyses included Cochran’s Q test, MR-Egger-intercept test, MR-PRESSO, and leave-one-out analysis to assess heterogeneity, horizontal pleiotropy, and robustness. To visualize the results, scatter plots, forest plots, leave-one-out plots, and funnel plots were generated. All statistical analyses were conducted using R software (Version 4.3.1). |
|  | b) | Describe how genetic variants were handled in the analyses and, if applicable, how their weights were selected | 10 | The selection of IVs followed three fundamental principles: the selected single nucleotide polymorphisms(SNPs) should be strongly associated with the exposure, influence the outcome only through the exposure, and remain independent of potential confounders.(37) To ensure compliance with these principles, we extracted cis-acting SNPs within a 500 kb region surrounding the DPP4 gene from the eQTL database, applying thresholds of P < 5×10⁻⁸, minor allele frequency (MAF) >1%, and false discovery rate (FDR) <0.05. To minimize linkage disequilibrium (LD) bias, independent SNPs were selected using LD clumping (r² < 0.3, 500kb). Additionally, the association between each DPP4 variant and glycated hemoglobin (HbA1c) levels was assessed, and SNPs with an F-statistic <10 was excluded to remove weak instrument bias. We also conducted a colocalization analysis between DPP4 and HbA1c, using a posterior probability threshold of >80% as evidence of colocalization. The final IVs obtained through this process were used as proxies for DPP4i. |
|  | c) | Describe the MR estimator (e.g. two-stage least squares, Wald ratio) and related statistics. Detail the included covariates and, in case of two-sample MR, whether the same covariate set was used for adjustment in the two samples | 10 | The product of coefficients method was used to estimate the mediation effect. In the MR analysis, the inverse-variance weighted (IVW) method was the primary analysis method, as it assumes that more than 50% of the IVs are valid. The MR-Egger regression and weighted median were used as supplementary methods. MR results were reported as odds ratio (OR) with corresponding 95% CI. |
|  | d) | Explain how missing data were addressed | NA | Used GWAS summary statistics |
|  | e) | If applicable, indicate how multiple testing was addressed | NA |  |
| 7 | **Assessment of assumptions** | Describe any methods or prior knowledge used to assess the assumptions or justify their validity | 10 | The product of coefficients method was used to estimate the mediation effect. In the MR analysis, the inverse-variance weighted (IVW) method was the primary analysis method, as it assumes that more than 50% of the IVs are valid. The MR-Egger regression and weighted median were used as supplementary methods. MR results were reported as odds ratio (OR) with corresponding 95% CI. Sensitivity analyses included Cochran’s Q test, MR-Egger-intercept test, MR-PRESSO, and leave-one-out analysis to assess heterogeneity, horizontal pleiotropy, and robustness. To visualize the results, scatter plots, forest plots, leave-one-out plots, and funnel plots were generated. All statistical analyses were conducted using R software (Version 4.3.1). |
| 8 | **Sensitivity analyses and additional analyses** | Describe any sensitivity analyses or additional analyses performed (e.g. comparison of effect estimates from different approaches, independent replication, bias analytic techniques, validation of instruments, simulations) | 10 | The product of coefficients method was used to estimate the mediation effect. In the MR analysis, the inverse-variance weighted (IVW) method was the primary analysis method, as it assumes that more than 50% of the IVs are valid. The MR-Egger regression and weighted median were used as supplementary methods. MR results were reported as odds ratio (OR) with corresponding 95% CI. Sensitivity analyses included Cochran’s Q test, MR-Egger-intercept test, MR-PRESSO, and leave-one-out analysis to assess heterogeneity, horizontal pleiotropy, and robustness. To visualize the results, scatter plots, forest plots, leave-one-out plots, and funnel plots were generated. All statistical analyses were conducted using R software (Version 4.3.1). |
| 9 | **Software and pre-registration** |  |  |  |
|  | a) | Name statistical software and package(s), including version and settings used | 10 | R software (Version 4.3.1). |
|  | b) | State whether the study protocol and details were pre-registered (as well as when and where) | NA | NA |
|  | **RESULTS** |  |  |  |
| 10 | **Descriptive data** |  |  |  |
|  | a) | Report the numbers of individuals at each stage of included studies and reasons for exclusion. Consider use of a flow diagram | NA | Used publicly available GWAS summary statistics |
|  | b) | Report summary statistics for phenotypic exposure(s), outcome(s), and other relevant variables (e.g. means, SDs, proportions) | 9 | Supplementary Table 5 |
|  | c) | If the data sources include meta-analyses of previous studies, provide the assessments of heterogeneity across these studies | NA | NA |
|  | d) | For two-sample MR:  i.  Provide justification of the similarity of the genetic variant-exposure associations between the exposure and outcome samples  ii.  Provide information on the number of individuals who overlap between the exposure and outcome studies | 9 | Supplementary Table 5 |
| 11 | **Main results** |  |  |  |
|  | a) | Report the associations between genetic variant and exposure, and between genetic variant and outcome, preferably on an interpretable scale | 17-18 | Based on the IVs selection criteria, 14 SNPs were identified as genetic proxies for DPP4i effects. All SNPs had F-statistics greater than 10, eliminating the influence of weak instruments. (Supplementary Table 9) The IVW model analysis demonstrated a causal association between DPP4i and RA, indicating that for each SD increase in DPP4i, the risk of RA decreased by 16% (OR = 0.84, 95% CI: 0.76–0.93). (Figure 6) The MR-Egger and weighted median yielded consistent results. (Supplementary Table 10) Sensitivity analyses detected no evidence of heterogeneity or horizontal pleiotropy (P values of Cochran Q test, MR-PRESSO and MR-Egger intercept test were all >0.05). (Supplementary Table 11) Leave-one-out analysis revealed that no single SNP had an obvious influence on the causal inference. (Supplementary Figure 7) |
|  | b) | Report MR estimates of the relationship between exposure and outcome, and the measures of uncertainty from the MR analysis, on an interpretable scale, such as odds ratio or relative risk per SD difference | 18-19 | We estimated the effects of 91 inflammatory cytokines and 731 immune cell types on RA and then identified 15 inflammatory cytokines and 11 immune cell types that exhibited a causal association with RA. Further analysis of the association between DPP4i and these inflammatory cytokines and immune cells revealed that DPP4i was causally associated with 8 inflammatory cytokines and 11 immune cell types. (Supplementary Table 12) Mediation effect analysis and mediation proportion calculations were conducted for the identified inflammatory cytokines and immune cells. The results indicated that 2 inflammatory cytokines and 4 immune cell types acted as mediators in the association between DPP4i and RA. (Figure 5) As shown in Table 2, DPP4i exerted an indirect effect on RA risk through CD14⁺ CD16⁺ monocyte AC, CD14⁺ CD16⁺ monocyte %monocyte, CD11b on CD14⁺ monocyte, CD62L⁻ HLA-DR⁺⁺ monocyte AC, C-X-C motif chemokine 11 (CXCL11) levels, and interleukin-2 receptor subunit beta (IL-2Rβ) levels. The proportions of the total effect mediated by these factors were 2%, 15%, 14%, 13%, 11%, and 10%, respectively. (Table 2) |
|  | c) | If relevant, consider translating estimates of relative risk into absolute risk for a meaningful time period | NA | NA |
|  | d) | Consider plots to visualize results (e.g. forest plot, scatterplot of associations between genetic variants and outcome versus between genetic variants and exposure) | Figure 6 and Supplementary Figure 7 | Figure 6 and Supplementary Figure 7 |
| 12 | **Assessment of assumptions** |  |  |  |
|  | a) | Report the assessment of the validity of the assumptions | Table 2,  Supplemental file Table S1-4 | This study used publicly available GWAS summary statistics which limits the assessment for testing the assumptions .The F statistic are in Supplemental file Table S1-4. |
|  | b) | Report any additional statistics (e.g., assessments of heterogeneity across genetic variants, such as I2, Q statistic or E-value) | 17 | Based on the IVs selection criteria, 14 SNPs were identified as genetic proxies for DPP4i effects. All SNPs had F-statistics greater than 10, eliminating the influence of weak instruments. (Supplementary Table 9) |
| 13 | **Sensitivity analyses and additional analyses** |  |  |  |
|  | a) | Report any sensitivity analyses to assess the robustness of the main results to violations of the assumptions | 17-18 | The MR-Egger and weighted median yielded consistent results. (Supplementary Table 10) Sensitivity analyses detected no evidence of heterogeneity or horizontal pleiotropy (P values of Cochran Q test, MR-PRESSO and MR-Egger intercept test were all >0.05). (Supplementary Table 11) Leave-one-out analysis revealed that no single SNP had an obvious influence on the causal inference. (Supplementary Figure 7) |
|  | b) | Report results from other sensitivity analyses or additional analyses | NA | NA |
|  | c) | Report any assessment of direction of causal relationship (e.g., bidirectional MR) | 17 | The IVW model analysis demonstrated a causal association between DPP4i and RA, indicating that for each SD increase in DPP4i, the risk of RA decreased by 16% (OR = 0.84, 95% CI: 0.76–0.93). (Figure 6) |
|  | d) | When relevant, report and compare with estimates from non-MR analyses | NA | NA |
|  | e) | Consider additional plots to visualize results (e.g., leave-one-out analyses) | Supplementary Figure 7 | Supplementary Figure 7 |
|  | **DISCUSSION** |  |  |  |
| 14 | **Key results** | Summarize key results with reference to study objectives | 20 | Our findings provided compelling evidence demonstrating that T2DM may be a risk factor for RA, and the protective effect of DPP4i on RA may be mediated through immune pathways. |
| 15 | **Limitations** | Discuss limitations of the study, taking into account the validity of the IV assumptions, other sources of potential bias, and imprecision. Discuss both direction and magnitude of any potential bias and any efforts to address them | 24-25 | Thirdly, this study evaluated the potential impact of DPP4i through its genetic proxies in MR analysis, which is based on genetically predicted DPP4i effects rather than actual drug use data. This limitation suggested that the findings may not fully reflect the real-world effects of clinical DPP4i use.  Nevertheless, variations in the type of DPP4i and treatment duration may exert distinct effects on RA, which could potentially influence our study findings. Fifthly, the study relied primarily on GWAS data to investigate the immune regulatory mechanisms of DPP4i. While we identified potential mediatory effects of specific immune cells and inflammatory cytokines, further cellular and animal model studies are required to directly validate the immunomodulatory effects of DPP4i and clarify its precise role in RA pathogenesis. |
| 16 | **Interpretation** |  |  |  |
|  | a) | Meaning: Give a cautious overall interpretation of results in the context of their limitations and in comparison with other studies | 24-25 | Thirdly, this study evaluated the potential impact of DPP4i through its genetic proxies in MR analysis, which is based on genetically predicted DPP4i effects rather than actual drug use data. This limitation suggested that the findings may not fully reflect the real-world effects of clinical DPP4i use.  Nevertheless, variations in the type of DPP4i and treatment duration may exert distinct effects on RA, which could potentially influence our study findings. Fifthly, the study relied primarily on GWAS data to investigate the immune regulatory mechanisms of DPP4i. While we identified potential mediatory effects of specific immune cells and inflammatory cytokines, further cellular and animal model studies are required to directly validate the immunomodulatory effects of DPP4i and clarify its precise role in RA pathogenesis. |
|  | b) | Mechanism: Discuss underlying biological mechanisms that could drive a potential causal relationship between the investigated exposure and the outcome, and whether the gene-environment equivalence assumption is reasonable. Use causal language carefully, clarifying that IV estimates may provide causal effects only under certain assumptions | 22-23 | Although multiple studies have investigated the anti-inflammatory and immunomodulatory effects of DPP4i, there have no experimental studies have specifically examined the mechanistic pathways linking DPP4i to RA. Therefore, we conducted a mediation MR analysis, which revealed that DPP4i might reduce RA risk by modulating specific immune cells and inflammatory cytokines. Studies have shown that CD14⁺CD16⁺ monocytes are highly enriched in RA synovial tissue and can promote the release of pro-inflammatory cytokines, such as TNF-α and IL-1β, thereby exacerbating RA-associated inflammatory responses.(53, 54) This study found that DPP4i can modulate the expression of CD14⁺CD16⁺ monocytes, suggesting that it may influence RA progression by regulating the infiltration of pro-inflammatory monocytes. Meanwhile, CXCL11, a chemokine significantly elevated in the serum of RA patients, plays a key role in promoting T cell and monocyte recruitment, thereby exacerbating synovial inflammation.(55-57) This study suggested that DPP4i might reduce RA risk by inhibiting CXCL11 expression, thereby limiting the infiltration of inflammatory cells and mitigating synovial inflammation. The IL-2/IL-2R plays a crucial role in immune regulation in RA, abnormal IL-2R expression may lead to excessive T-cell activation, thereby exacerbating RA-associated immunopathology.(58) This study further demonstrated that DPP4i might modulate IL-2R signaling, potentially contributing to the restoration of immune balance in RA. These research results indicated that DPP4i might play a role in RA immune regulation, providing a new research direction for the potential therapeutic applications of DPP4i in autoimmune diseases. |
|  | c) | Clinical relevance: Discuss whether the results have clinical or public policy relevance, and to what extent they inform effect sizes of possible interventions | 23 | These research results indicated that DPP4i might play a role in RA immune regulation, providing a new research direction for the potential therapeutic applications of DPP4i in autoimmune diseases. |
| 17 | **Generalizability** | Discuss the generalizability of the study results (a) to other populations, (b) across other exposure periods/timings, and (c) across other levels of exposure | 24-25 | Thirdly, this study evaluated the potential impact of DPP4i through its genetic proxies in MR analysis, which is based on genetically predicted DPP4i effects rather than actual drug use data. This limitation suggested that the findings may not fully reflect the real-world effects of clinical DPP4i use.  Nevertheless, variations in the type of DPP4i and treatment duration may exert distinct effects on RA, which could potentially influence our study findings. Fifthly, the study relied primarily on GWAS data to investigate the immune regulatory mechanisms of DPP4i. While we identified potential mediatory effects of specific immune cells and inflammatory cytokines, further cellular and animal model studies are required to directly validate the immunomodulatory effects of DPP4i and clarify its precise role in RA pathogenesis. |
|  | **OTHER INFORMATION** |  |  |  |
| 18 | **Funding** | Describe sources of funding and the role of funders in the present study and, if applicable, sources of funding for the databases and original study or studies on which the present study is based | 25 | This work was supported by the Natural Science Foundation of Fujian, China [grant number 2021J01397]; the Fujian provincial health technology project [grant number 2022GGA010]; the Fujian provincial Joint Funding Project of Scientific and Technological Innovation [grant numbers 2023Y9347]. All authors acknowledge that they had access to all study data and are responsible for the decision to submit for publication. |
| 19 | **Data and data sharing** | Provide the data used to perform all analyses or report where and how the data can be accessed, and reference these sources in the article. Provide the statistical code needed to reproduce the results in the article, or report whether the code is publicly accessible and if so, where | NA | Used publicly available GWAS summary statistics |
| 20 | **Conflicts of Interest** | All authors should declare all potential conflicts of interest | 25 | The authors have no relevant financial or non-financial interests to disclose. |

This checklist is copyrighted by the Equator Network under the Creative Commons Attribution 3.0 Unported (CC BY 3.0) license.

1. Skrivankova VW, Richmond RC, Woolf BAR, Yarmolinsky J, Davies NM, Swanson SA, et al. Strengthening the Reporting of Observational Studies in Epidemiology using Mendelian Randomization (STROBE-MR) Statement. JAMA. 2021;under review.

2. Skrivankova VW, Richmond RC, Woolf BAR, Davies NM, Swanson SA, VanderWeele TJ, et al. Strengthening the Reporting of Observational Studies in Epidemiology using Mendelian Randomisation (STROBE-MR): Explanation and Elaboration. BMJ. 2021;375:n2233.

**Supplementary Table 1** Overview of epidemiological studies investigating the associations between T2DM or DPP4i and RA.

| Authors (Published year) | Type of Research | Samplesize | Findings |
| --- | --- | --- | --- |
| Su CC et al (2013) | Retrospective study | 600,695 | The RA to non-RA risk ratio for T2DM was 1.68 (95% CI 1.53-1.84) in men and 1.46 (95% CI: 1.39-1.54) in women |
| Jin YZ et al (2020) | Retrospective study | 217,136 | RA was associated with a 24-35% lower risk of incident T2DM compared to 4 comparison groups |
| Pei RM et al (2024) | Retrospective study | 694 | The incidence of RA in patients with T2DM was 15.6% |
| Zhang P et al (2024) | Observational + MR hybrid study | 300,62 | RA remained significant association with diabetes (OR = 1.12; 95% CI: 1.02–1.32) |
| Yokota K et al (2012) | Case report | 1 | Sitagliptin (DPP4i)-induced RA in type 2 diabetes mellitus |
| NCT01107886(2014) | RCT | 16,492 | Two case of RA was reported in the placebo group and none in the saxagliptin group |
| NCT00968708 (2014) | RCT | 5,380 | One case of RA was reported in the placebo group and none in the alogliptin group |
| NCT00121667 (2015) | RCT | 551 | Two case of RA was reported in the saxagliptin group and none in the placebo group |
| NCT01652729 (2015) | RCT | 303 | No case of RA was reported in the sitagliptin group and one in the placebo group |
| Kim SC et al (2015) | Retrospective study | 739,28 | The risk of incident RA (HR=0.66, 95% CI: 0.44–0.99) within 365 days of follow-up was decreased for DPP4i initiators compared to non-DPP4i |
| Douros A et al (2018) | Retrospective study | 144,603 | Compared with use of other antidiabetic drugs, use of DPP4i was not associated with an increased risk of RA (HR = 1.0; 95% CI: 0.8, 1.3) |
| Khalaf Kridin et al (2018) | Retrospective study | 5,943 | The prevalence of the RA did not differ significantly between DPP4i-treated patients and their matched control subjects |
| Seong JM et al (2019) | Retrospective study | 114,078,4 | The risk of incident RA was decreased for DPP4i initiators compared with non-DPP4i initiators (aHR=0.72, 95% CI: 0.51-1.01) |
| NCT01897532 (2019) | RCT | 6,979 | One case of RA was reported in the linagliptin group and none in the placebo group |
| NCT01243424 (2020) | RCT | 6033 | No case of RA was reported in the linagliptin group and two in the placebo group |
| Yi-Chuan Chen et al (2020) | Retrospective study | 74,198 | DPP-4 inhibitors are associated with lower risk of autoimmune disorders in type 2 diabetes mellitus patients in Taiwan |
| NCT00790205 (2021) | RCT | 14,540 | One case of RA was reported in the sitagliptin group and one in the placebo group |
| Tomoyuki Katsuno et al (2021) | RCT | 245 | No case of RA was reported in the linagliptin group and one in the placebo group |

**Supplementary Table 2** The definitions of relevant variables from the NHANES database used in this study.

| Concomitant variable | Grading |
| --- | --- |
| Gender | Male and female |
| Age | <40, 41–60, 61–80, and >80 years |
| Race | Hispanic and non-Hispanic |
| Educational level | less than high school, high school or general educational development, and above high school |
| BMI | underweight (BMI <18.5 kg/m²), normal weight (18.5 ≤ BMI < 25 kg/m²), overweight (25 ≤ BMI < 29.9 kg/m²), and obesity (BMI ≥ 30 kg/m²) |
| Health insurance status | Insured and uninsured |
| Drinking | consuming ≥5 alcoholic drinks per day |
| Smoking | having smoked at least 100 cigarettes in a lifetime |
| Hypertension | having been diagnosed with high blood pressure by a physician |

**Supplementary Table 3** The search strategy for the meta-analysis conducted in this study.

| Database | No. | Query Results | Result |
| --- | --- | --- | --- |
| Pubmed | #1 | (Arthritis, Rheumatoid [MeSH Terms]) AND (Rheumatoid Arthritis) | 130853 |
|  | #2 | (((((((((((((((((((((((((((((((((Dipeptidyl-Peptidase IV Inhibitors[MeSH Terms]) OR (DPP-4 Inhibitor[Title/Abstract])) OR (DPP-IV Inhibitor[Title/Abstract])) OR (DPP 4 Inhibitor[Title/Abstract])) OR (Inhibitor, DPP-4[Title/Abstract])) OR (DPP IV Inhibitor[Title/Abstract])) OR (Inhibitor, DPP-IV[Title/Abstract])) OR (DPP-4 Inhibitors[Title/Abstract])) OR (DPP 4 Inhibitors[Title/Abstract])) OR (DPP-IV Inhibitors[Title/Abstract])) OR (DPP IV Inhibitors[Title/Abstract])) OR (DPP4 Inhibitors[Title/Abstract])) OR (Dipeptidyl Peptidase 4 Inhibitor[Title/Abstract])) OR (Dipeptidyl-Peptidase IV Inhibitor[Title/Abstract])) OR (Dipeptidyl Peptidase IV Inhibitor[Title/Abstract])) OR (Inhibitor, Dipeptidyl-Peptidase IV[Title/Abstract])) OR (Dipeptidyl-Peptidase 4 Inhibitor[Title/Abstract])) OR (inhibitor, Dipeptidyl-Peptidase 4[Title/Abstract])) OR (Dipeptidyl-Peptidase 4 Inhibitors[Title/Abstract])) OR (Dipeptidyl Peptidase 4 Inhibitors[Title/Abstract])) OR (Gliptins[Title/Abstract])) OR (DPP4 Inhibitor[Title/Abstract])) OR (Gliptin[Title/Abstract])) OR (sitagliptin[Title/Abstract])) OR (vildagliptin[Title/Abstract])) OR (omarigliptin[Title/Abstract])) OR (saxagliptin[Title/Abstract])) OR (alogliptin[Title/Abstract])) OR (trelagliptin[Title/Abstract])) OR (anagliptin[Title/Abstract])) OR (linagliptin[Title/Abstract])) OR (gemigliptin[Title/Abstract])) OR (evogliptin[Title/Abstract])) OR (teneligliptin[Title/Abstract]) | 11933 |
|  | #3 | #1 AND #2 | 16 |
| Web of Science | #1 | ((TS=(Arthritis, Rheumatoid)) OR TS=(Rheumatoid Arthritis)) | 325697 |
|  | #2 | ((((((((((((((((((((((((((((((((((TS=(Dipeptidyl-Peptidase IV Inhibitors)) OR TS=(DPP-4 Inhibitor、DPP 4 Inhibitor)) OR TS=(Inhibitor, DPP-4)) OR TS=(DPP-IV Inhibitor)) OR TS=(DPP IV Inhibitor)) OR TS=(Inhibitor, DPP-IV)) OR TS=(DPP-4 Inhibitors)) OR TS=(DPP 4 Inhibitors)) OR TS=(DPP-IV Inhibitors)) OR TS=(DPP IV Inhibitors)) OR TS=(DPP4 Inhibitors)) OR TS=(Dipeptidyl Peptidase 4 Inhibitor)) OR TS=(Dipeptidyl-Peptidase IV Inhibitor)) OR TS=(Dipeptidyl Peptidase IV Inhibitor)) OR TS=(Inhibitor, Dipeptidyl-Peptidase IV)) OR TS=(Dipeptidyl-Peptidase 4 Inhibitor)) OR TS=(Inhibitor, Dipeptidyl-Peptidase 4)) OR TS=(Dipeptidyl-Peptidase 4 Inhibitors)) OR TS=(Dipeptidyl Peptidase 4 Inhibitors)) OR TS=(DPP4 Inhibitor)) OR TS=(Inhibitor, DPP4)) OR TS=(DPP4i)) OR TS=(Gliptins)) OR TS=(Gliptin)) OR TS=(sitagliptin)) OR TS=(vildagliptin)) OR TS=(omarigliptin)) OR TS=(saxagliptin)) OR TS=(alogliptin)) OR TS=(trelagliptin)) OR TS=(anagliptin)) OR TS=(linagliptin)) OR TS=(gemigliptin)) OR TS=(evogliptin)) OR TS=(teneligliptin) | 22239 |
|  | #3 | #1 AND #2 | 220 |
| Cochrane | #1 | (Arthritis, Rheumatoid or Rheumatoid Arthritis):ti,ab,kw | 19597 |
|  | #2 | (Dipeptidyl-Peptidase IV Inhibitors or DPP-4 Inhibitor or DPP 4 Inhibitor or Inhibitor, DPP-4 or DPP-IV Inhibitor or DPP IV Inhibitor or Inhibitor, DPP-IV or DPP-4 Inhibitors or DPP 4 Inhibitors or DPP-IV Inhibitors or DPP IV Inhibitors or DPP4 Inhibitors or Dipeptidyl Peptidase 4 Inhibitor or Dipeptidyl-Peptidase IV Inhibitor or Dipeptidyl Peptidase IV Inhibitor or Inhibitor, Dipeptidyl-Peptidase IV or Dipeptidyl-Peptidase 4 Inhibitor or Inhibitor, Dipeptidyl-Peptidase 4 or Dipeptidyl-Peptidase 4 Inhibitors or Dipeptidyl Peptidase 4 Inhibitors or DPP4 Inhibitor or Inhibitor, DPP4 or DPP4i or Gliptins or Gliptin or sitagliptin or vildagliptin or omarigliptin or saxagliptin or alogliptin or trelagliptin or anagliptin or linagliptin or gemigliptin or evogliptin or teneligliptin):ti,ab,kw | 5901 |
|  | #3 | #1 AND #2 | 4 |
| Embase | #1 | rheumatoid arthritis' OR 'arthritis, rheumatoid' | 298110 |
|  | #2 | Dipeptidyl-Peptidase IV Inhibitors' OR 'DPP-4 Inhibitor' OR 'DPP 4 Inhibitor' OR 'Inhibitor, DPP-4'OR 'DPP-IV Inhibitor' OR 'DPP IV Inhibitor'OR 'Inhibitor, DPP-IV' OR 'DPP-4 Inhibitors' OR 'DPP 4 Inhibitors' OR 'DPP-IV Inhibitors' OR 'DPP IV Inhibitors' OR 'DPP4 Inhibitors' OR 'Dipeptidyl Peptidase 4 Inhibitor' OR 'Dipeptidyl-Peptidase IV Inhibitor' OR 'Dipeptidyl Peptidase IV Inhibitor' OR 'Inhibitor, Dipeptidyl-Peptidase IV' OR 'Dipeptidyl-Peptidase 4 Inhibitor'OR 'Inhibitor, Dipeptidyl-Peptidase 4' OR 'Dipeptidyl-Peptidase 4 Inhibitors' OR 'Dipeptidyl Peptidase 4 Inhibitors' OR 'DPP4i' OR 'Gliptins' OR 'DPP4 Inhibitor' OR 'Inhibitor, DPP4' OR 'sitagliptin'OR 'vildagliptin' OR 'omarigliptin'OR 'saxagliptin' OR 'alogliptin' OR 'trelagliptin' OR 'anagliptin' OR 'linagliptin' OR 'gemigliptin' OR 'evogliptin' OR 'teneligliptin' | 11022 |
|  | #3 | #1 AND #2 | 28 |
| Scopus | #1 | (TITLE-ABS-KEY ( rheumatoid AND arthritis ) OR TITLE-ABS-KEY ( arthritis, AND rheumatoid ) ) | 244493 |
|  | #2 | ( TITLE-ABS-KEY ( dipeptidyl-peptidase AND iv AND inhibitors ) OR TITLE-ABS-KEY ( dpp-4 AND inhibitor ) OR TITLE-ABS-KEY ( dpp4 AND inhibitor ) OR TITLE-ABS-KEY ( dpp-iv AND inhibitor ) OR TITLE-ABS-KEY ( inhibitor AND dpp-4 ) OR TITLE-ABS-KEY ( inhibitor AND dpp-iv ) OR TITLE-ABS-KEY ( dpp-4 AND inhibitors ) OR TITLE-ABS-KEY ( dpp4 AND inhibitors ) OR TITLE-ABS-KEY ( dpp-iv AND inhibitors ) OR TITLE-ABS-KEY ( dipeptidyl AND peptidase 4 inhibitor ) OR TITLE-ABS-KEY ( inhibitor AND dipeptidyl-peptidase AND iv ) OR TITLE-ABS-KEY ( inhibitor AND dipeptidyl-peptidase 4 ) OR TITLE-ABS-KEY ( dipeptidyl-peptidase 4 inhibitors ) OR TITLE-ABS-KEY ( dipeptidyl AND peptidase 4 inhibitors ) OR TITLE-ABS-KEY ( gliptins ) OR TITLE-ABS-KEY ( gliptin ) OR TITLE-ABS-KEY ( sitagliptin ) OR TITLE-ABS-KEY ( vildagliptin ) OR TITLE-ABS-KEY ( omarigliptin ) OR TITLE-ABS-KEY ( saxagliptin ) OR TITLE-ABS-KEY ( alogliptin ) OR TITLE-ABS-KEY ( trelagliptin ) OR TITLE-ABS-KEY ( anagliptin ) OR TITLE-ABS-KEY ( linagliptin ) OR TITLE-ABS-KEY ( gemigliptin ) OR TITLE-ABS-KEY ( evogliptin ) OR TITLE-ABS-KEY ( teneligliptin ) ) | 28243 |
|  | #3 | #1 AND #2 | 284 |
| Cochrane Central Register of Controlled Trials | #1 | Dipeptidyl-Peptidase IV Inhibitors | 422 |

**Supplementary Table 4** Inclusion and exclusion criteria for references

| Inclusion criteria: |
| --- |
| (1) The subjects were patients with T2DM  (2) The intervention group was T2DM patients treated with DPP4i, and the control group was T2DM patients not treated with DPP4i  (3) The study outcome reported whether RA occurred, and the number of RA events reported  (4) The study type was RCT or cohort study |
| Exclusion criteria: |
| (1) The subjects were T1DM, had other types of autoimmune diseases or pregnant woman  (2) The study type was not an RCTs or cohort study  (3) Studies at the cellular or animal level  (4) Meta-analysis or systematic review  (5) Letters, comments, meeting reports or republished literature  (6) The reported data is incomplete |

**Supplementary Table 5** Detailed information for genome-wide association study (GWAS) statistics used in the present study.

| Trait | Souces | ID | Samplesize |
| --- | --- | --- | --- |
| DPP4 | eQTLGen Consortium | --- | 31684 |
| HbA1c | IEU | ukb-d-30750_irnt | 344182 |
| RA | IEU | ebi-a-GCST90013534 | 58284 |
| T2DM | FinnGen | --- | 440735 |
| Immune cell | GWAS catalog | GCST90001902 | 3757 |
| Inflammatory factor | GWAS catalog | GCST90274797 | 14824 |

**Supplementary Table 6** Baseline characteristics of RA group versus the non-RA arthritis group.

| Characteristics | Overall | RA | Non-RA | P value |
| --- | --- | --- | --- | --- |
| Age(years) | 47.64±0.2292 | 60.21±0.28 | 43.58±0.22 | <0.0001 |
| Gender (%) |  |  |  | <0.0001 |
| Male | 6572 (0.42) | 1517 (0.36) | 5055 (0.44) |  |
| Female | 8490 (0.58) | 2634 (0.64) | 5856 (0.56) |  |
| Race (%) |  |  |  | <0.0001 |
| Hispanic | 336 (0.12) | 700 (0.07) | 2636 (0.13) |  |
| Non-Hispanic | 11726 (0.88) | 3451 (0.93) | 8275 (0.87) |  |
| Education level (%) |  |  |  | <0.0001 |
| Less than high school | 3278 (0.13) | 1016 (0.15) | 2262 (0.12) |  |
| High school or GED | 3108 (0.21) | 933 (0.24) | 2175 (0.20) |  |
| Above high school | 8676 (0.66) | 2202 (0.61) | 6474 (0.68) |  |
| Health Insurance |  |  |  | <0.0001 |
| Yes | 12346 (0.85) | 3787 (0.92) | 8559 (0.83) |  |
| No | 2716 (0.15) | 364 (0.08) | 2352 (0.17) |  |
| Smoking |  |  |  | <0.0001 |
| Yes | 6772 (0.46) | 2183 (0.54) | 4589 (0.43) |  |
| No | 8290 (0.54) | 1968 (0.46) | 6322 (0.57) |  |
| Alcohol (%) |  |  |  | 0.003 |
| Yes | 2432 (0.15) | 773 (0.17) | 1659 (0.15) |  |
| No | 12630 (0.85) | 3378 (0.83) | 9252 (0.85) |  |
| BMI (kg/m2) | 28.76±0.09 | 30.71±0.17 | 28.12±0.09 | <0.0001 |
| T2DM (%) |  |  |  | <0.0001 |
| Yes | 1456 (0.07) | 728 (0.14) | 728 (0.05) |  |
| No | 13606 (0.93) | 3423 (0.86) | 10183 (0.95) |  |
| Hypertension (%) |  |  |  | <0.0001 |
| Yes | 5161 (0.30) | 2344 (0.52) | 2817 (0.23) |  |
| No | 9901 (0.70) | 1807 (0.48) | 8094 (0.77) |  |

Abbreviations: GED, General Educational Development; BMI, body mass index; T2DM, Type 2 diabetes mellitus; RA, Rheumatoid arthritis

**Supplementary Table 7** Description of included studies

| Study | Region | Study design | Age(year) | Male,No.（%） | Samplesize | Duration of follow-up (years) | Type of DPP4i |
| --- | --- | --- | --- | --- | --- | --- | --- |
| NCT01107886 2014 | Multiple regions | RCT | Experimental group：65.0 Control group：65.1 | Experimental group：66.6 Control group：67.3 | 16492 | Up to 2.9 years | Saxagliptin |
| NCT00121667 2015 | Multiple regions | RCT | Experimental group：54.7 Control group：54.8 | Experimental group：53.2 Control group：53.6 | 551 | Up to 3.7 years | Saxagliptin |
| NCT01652729 2015 | Multiple regions | RCT | Experimental group：54.3 Control group：53.4 | Experimental group：54.1 Control group：60.7 | 303 | Up to 7 months | Sitagliptin |
| NCT01897532 2019 | Multiple regions | RCT | Experimental group：66.1 Control group：65.6 | Experimental group：61.5 Control group：64.3 | 6979 | Up to 4.3 years | Linagliptin |
| NCT01243424 2020 | Multiple regions | RCT | Experimental group：63.9 Control group：64.2 | Experimental group：60.8 Control group：59.2 | 6033 | Up to 8.3 years | Linagliptin |
| Jong-Mi Seong 2019 | Korea | Cohort study | Experimental group：57.7 Control group：57.7 | Experimental group：57.9 Control group：58.5 | 1140784 | 1.68 year | Multiple DPP4i |
| Khalaf Kridin 2018 | Israel | Cohort study | Experimental group：74.5 Control group：74.4 | Experimental group：43.8 Control group：43.8 | 5943 | Up to 8 years | Multiple DPP4i |
| NCT00790205 2021 | Multiple regions | RCT | ___ | Experimental group：70.9 Control group：70.5 | 14540 | Up to 5 years | Sitagliptin |
| Seoyoung C Kim 2015 | USA | Cohort study | Experimental group：55.5 Control group：55.4 | Experimental group：60.0 Control group：61.0 | 95768 | Experimental group：0.72 year Control group：0.74 year | Multiple DPP4i |
| NCT00968708 2014 | Multiple regions | RCT | Experimental group：61.0 Control group：60.7 | Experimental group：61.3 Control group：60.3 | 5380 | Up to 3.4 years | Alogliptin |
| Tomoyuki Katsuno 2021 | Multiple regions | RCT | Experimental group：64.3 Control group：65.2 | Experimental group：61.5 Control group：61.3 | 245 | Up to 6 years | Linagliptin |
| Yi-Chuan Chen 2020 | China | Cohort study | Experimental group：60.2 Control group：60.1 | Experimental group：52.1 Control group：53.3 | 74198 | Up to 4 years | Multiple DPP4i |

**Supplementary Table 8** NOS for quality assessment of included cohort studies

| Study | Jong-Mi Seong 2019 | Khalaf Kridin 2018 | Seoyoung C Kim 2015 | Yi-Chuan Chen 2020 |
| --- | --- | --- | --- | --- |
| Representativeness of the exposed cohort | 1 | 1 | 1 | 1 |
| Selection of the non-exposed cohort | 1 | 1 | 1 | 1 |
| Ascertainment of exposure | 1 | 1 | 1 | 1 |
| Demonstration that outcome of interest was not present at start of study | 1 | 2 | 2 | 2 |
| Comparability of cohorts on basis of the design or analysis | 1 | 1 | 1 | 1 |
| Assessment of outcome | 1 | 1 | 1 | 1 |
| Whether the follow-up period was more than 0.25 years | 1 | 1 | 1 | 1 |
| Adequacy of follow up of cohorts | 0 | 0 | 1 | 0 |
| Final score | 7 | 8 | 9 | 8 |

**Supplementary Table 9** The F-statistics of the IVs for DPP4i used in this study.

| SNP | Effect_allele.exposure | Other_allele.exposure | Eaf.exposure | Pval.exposure | R2 | Fvalue |
| --- | --- | --- | --- | --- | --- | --- |
| rs12617336 | C | G | 0.016259629 | 4.14E-23 | 0.003498155 | 98.01486013 |
| rs13015258 | T | G | 0.566372207 | 8.90E-56 | 0.008098598 | 247.5298384 |
| rs145605013 | G | T | 0.039976526 | 3.69E-10 | 0.001486896 | 39.27231097 |
| rs183705644 | G | A | 0.038210605 | 3.54E-08 | 0.00104169 | 30.38336546 |
| rs2216447 | C | T | 0.316051978 | 3.65E-15 | 0.001976786 | 61.87314131 |
| rs2268889 | T | C | 0.353057236 | 3.38E-12 | 0.001527046 | 48.45385473 |
| rs2389643 | T | C | 0.87824974 | 3.66E-17 | 0.002353515 | 70.94894877 |
| rs2909445 | A | G | 0.199543189 | 1.12E-16 | 0.00217326 | 68.73964116 |
| rs35733101 | C | T | 0.212522998 | 6.43E-14 | 0.001866212 | 56.23126132 |
| rs67264085 | C | T | 0.300676142 | 1.60E-25 | 0.004049513 | 109.025143 |
| rs75062246 | C | T | 0.816634669 | 8.27E-28 | 0.003956471 | 119.463514 |
| rs75166367 | A | G | 0.062929599 | 2.84E-14 | 0.002025738 | 57.84261241 |
| rs7564465 | A | G | 0.55145197 | 3.61E-09 | 0.001146027 | 34.82068692 |
| rs7566261 | G | A | 0.259988584 | 1.61E-21 | 0.002886479 | 90.76175094 |

**Supplementary Table 10** The results of MR-Egger and weighted median methods evaluating the association between DPP4i and RA.

| Outcome | Exposure | Method | nSNP | **Beta** | Se | Pval | Or | Or_lci95 | Or_uci95 |
| --- | --- | --- | --- | --- | --- | --- | --- | --- | --- |
| Rheumatoid arthritis | DPP4i | MR Egger | 14 | -0.068 | 0.110 | 0.045 | 0.934 | 0.753 | 1.159 |
| Rheumatoid arthritis | DPP4i | Weighted median | 14 | -0.170 | 0.074 | 0.022 | 0.844 | 0.729 | 0.976 |
| Rheumatoid arthritis | DPP4i | Inverse variance weighted | 14 | -0.176 | 0.053 | 0.001 | 0.839 | 0.755 | 0.931 |

**Supplementary Table 11** The sensitivity analysis results of the MR study.

| Exposure | Outcome | Q_pval | Egger_intercept | MR-Egger_intercept P value | MR-PRESSO |
| --- | --- | --- | --- | --- | --- |
| DPP4i | Rheumatoid arthritis | 0.875959765 | 0.012992617 | 0.284369497 | 0.832 |

**Supplementary Table 12** The Beta and standard error (Se) values in the MR process for inflammatory factors and immune cells that have a causal association with DPP4i and RA.

| Trait | Beta1 | Se1 | P1 | Beta2 | Se2 | P2 |
| --- | --- | --- | --- | --- | --- | --- |
| CD11c+ monocyte AC | 0.2575218 | 0.1147396 | 0.02480654 | -0.079089155 | 0.03615861 | 0.028721785 |
| CD62L- HLA DR++ monocyte AC | 0.3619674 | 0.1138822 | 0.001480712 | -0.063877157 | 0.031267018 | 0.041056748 |
| CD14+ CD16+ monocyte AC | 0.4151746 | 0.1073538 | 0.000110025 | -0.088306402 | 0.030464053 | 0.003747034 |
| CD14+ CD16+ monocyte %monocyte | 0.2443752 | 0.1086507 | 0.02450095 | -0.110708155 | 0.030713021 | 0.000312634 |
| CD8br AC | 0.2319693 | 0.104794 | 0.02685806 | 0.05377738 | 0.022382876 | 0.016278423 |
| CD27 on IgD+ CD24+ | -0.238501 | 0.1095006 | 0.02940009 | -0.077091947 | 0.030807099 | 0.012335149 |
| CCR7 on naive CD4+ | -0.2668838 | 0.1232523 | 0.03036128 | 0.040784722 | 0.018446065 | 0.027034064 |
| CCR2 on monocyte | -0.2592829 | 0.1213125 | 0.03257245 | -0.075961984 | 0.027185367 | 0.005202455 |
| CD11b on CD14+ monocyte | 0.40538595 | 0.17474 | 0.02034421 | -0.062898222 | 0.016119039 | 9.54E-05 |
| HLA DR on B cell | -0.349478532 | 0.1177904 | 0.003007668 | -0.143168742 | 0.045584014 | 0.001685063 |
| CD8 on CD39+ CD8br | 0.24002752 | 0.1179078 | 0.04177819 | -0.080886473 | 0.035344352 | 0.022106902 |
| beta-nerve growth factor levels | 0.15365567 | 0.044690295 | 0.000585523 | 0.119385278 | 0.04828756 | 0.013421614 |
| T-cell surface glycoprotein CD5 levels | 0.190319899 | 0.043391979 | 1.15E-05 | 0.19735397 | 0.02924749 | 1.50E-11 |
| Macrophage colony-stimulating factor 1 levels | 0.159854115 | 0.04428859 | 0.000306936 | 0.134747258 | 0.067543894 | 0.046047417 |
| C-X-C motif chemokine 11 levels | -0.126736868 | 0.049239078 | 0.010055694 | 0.149247756 | 0.054783544 | 0.006443458 |
| Protein S100-A12 levels | -0.151772292 | 0.043876509 | 0.000542027 | -0.140025584 | 0.051461305 | 0.006508716 |
| Interleukin-2 receptor subunit beta levels | 0.120384002 | 0.048986292 | 0.01399063 | -0.143326387 | 0.067054828 | 0.032561355 |
| Macrophage inflammatory protein 1a levels | 0.11482474 | 0.043512648 | 0.008317994 | 0.140956145 | 0.03663511 | 0.000119295 |
| Signaling lymphocytic activation molecule levels | 0.12202997 | 0.047254801 | 0.009812078 | 0.160642902 | 0.057651634 | 0.005329025 |

Abbreviations: Beta1 and Se1 represent the beta and se values in the MR analysis of the association between DPP4i and inflammatory factors or immune cells；Beta2 and Se2 represent the beta and se values in the MR analysis of the association between inflammatory factors or immune cells and RA.


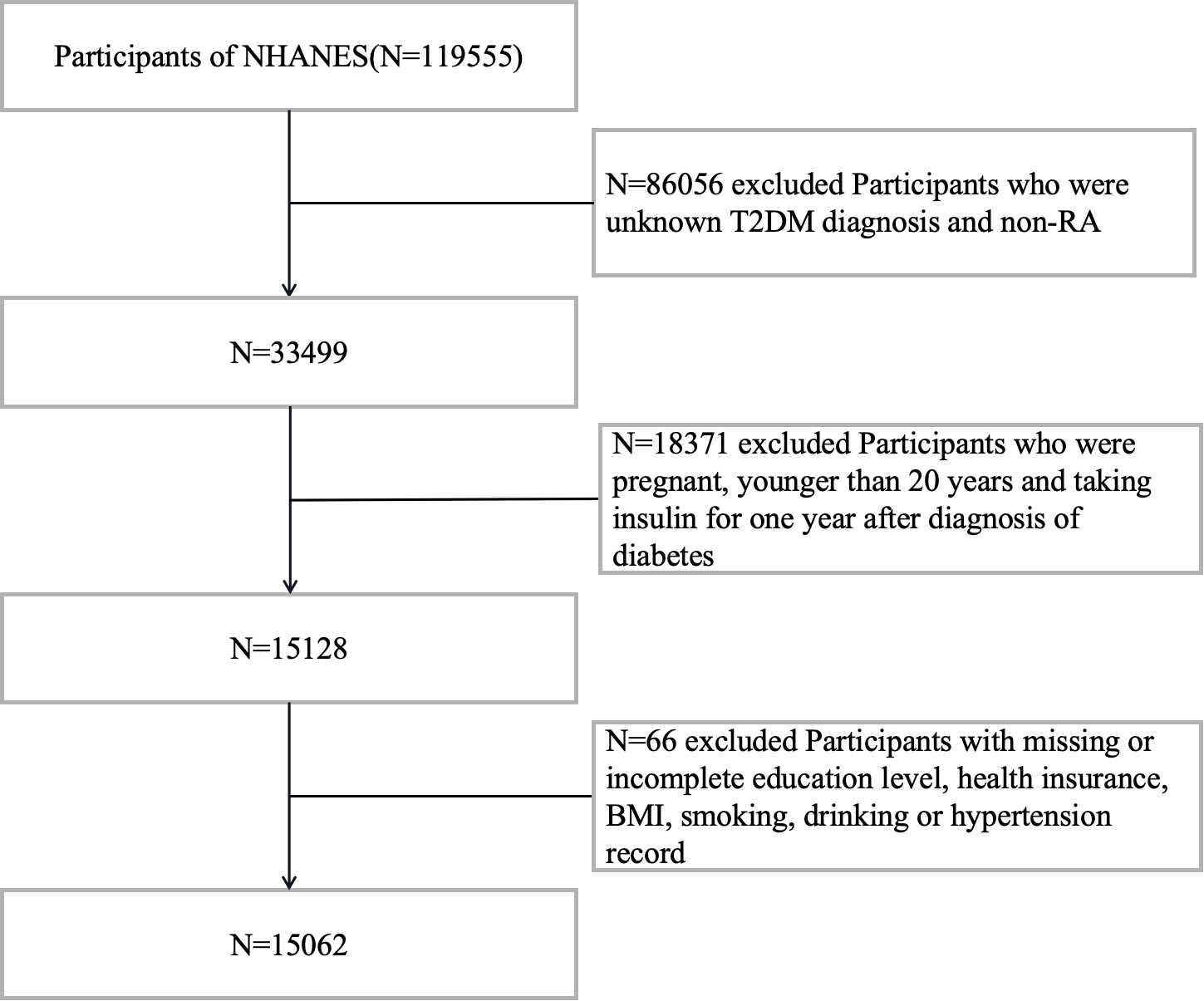


**Supplementary Figure 1** Flowchart of the selection and screening process for NHANES eligible participants from 1999 to 2023.


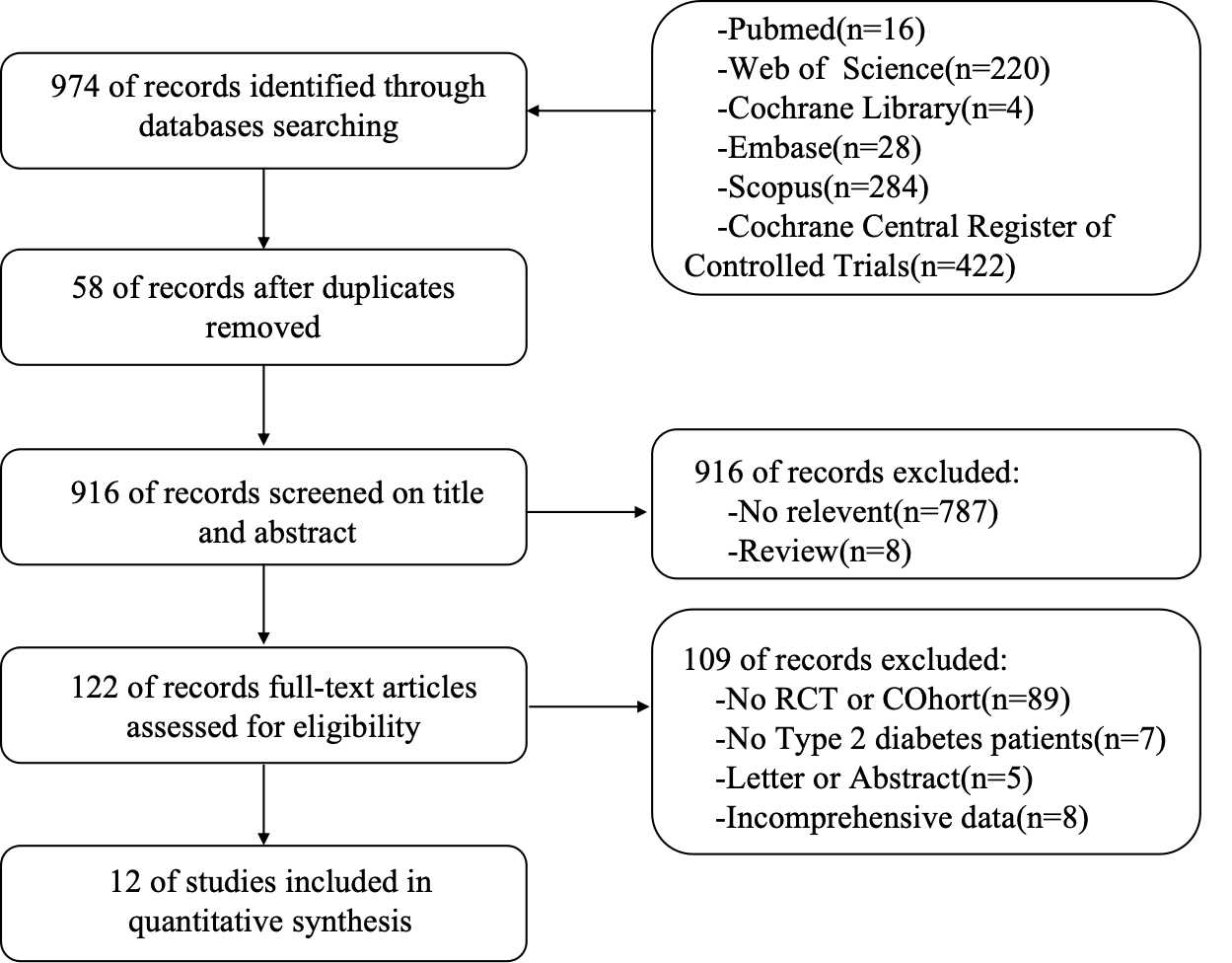


**Supplementary Figure 2** Flow diagram of the literature search and selection criteria

**Supplementary Figure 3 Bias analysis of the included RCTs literature**

**Supplementary Figure 4 Evaluation of the quality of the included RCTs literature**

**Supplementary Figure 5 The funnel plot of included studies**

**Supplementary Figure 6 The result of sensitivity analysis**

**Supplementary Figure 7 Sensitivity analysis results of MR study**
